# Distribution of the SARS-CoV-2 Pandemic and Its Monthly Forecast Based on Seasonal Climate Patterns

**DOI:** 10.3390/ijerph17103493

**Published:** 2020-05-17

**Authors:** Nicola Scafetta

**Affiliations:** Department of Earth Sciences, Environment and Georesources, University of Naples Federico II, Via Cinthia 21, 80126 Naples, Italy; nicola.scafetta@unina.it; Tel.: +39-081-2538348

**Keywords:** SARS-CoV-2, COVID-19, pandemic geographical distribution, epidemic forecasting, weather conditions, climatic zones, air pollution, population median age

## Abstract

This paper investigates whether the Severe Acute Respiratory Syndrome CoronaVirus 2 (SARS-CoV-2) pandemic could have been favored by specific weather conditions and other factors. It is found that the 2020 winter weather in the region of Wuhan (Hubei, Central China)—where the virus first broke out in December and spread widely from January to February 2020—was strikingly similar to that of the Northern Italian provinces of Milan, Brescia and Bergamo, where the pandemic broke out from February to March. The statistical analysis was extended to cover the United States of America, which overtook Italy and China as the country with the highest number of confirmed COronaVIrus Disease 19 (COVID-19) cases, and then to the entire world. The found correlation patterns suggest that the COVID-19 lethality significantly worsens (4 times on average) under weather temperatures between 4 °C and 12 °C and relative humidity between 60% and 80%. Possible co-factors such as median population age and air pollution were also investigated suggesting an important influence of the former but not of the latter, at least, on a synoptic scale. Based on these results, specific isotherm world maps were generated to locate, month by month, the world regions that share similar temperature ranges. From February to March, the 4–12 °C isotherm zone extended mostly from Central China toward Iran, Turkey, West-Mediterranean Europe (Italy, Spain and France) up to the United State of America, optimally coinciding with the geographic regions most affected by the pandemic from February to March. It is predicted that in the spring, as the weather gets warm, the pandemic will likely worsen in northern regions (United Kingdom, Germany, East Europe, Russia and North America) while the situation will likely improve in the southern regions (Italy and Spain). However, in autumn, the pandemic could come back and affect the same regions again. The Tropical Zone and the entire Southern Hemisphere, but in restricted colder southern regions, could avoid a strong pandemic because of the sufficiently warm weather during the entire year and because of the lower median age of their population. Google-Earth-Pro interactive-maps covering the entire world are provided as supplementary files.

## 1. Introduction

The Severe Acute Respiratory Syndrome CoronaVirus 2 (SARS-CoV-2) pneumonia—also known as COronaVIrus Disease 19 (COVID-19)—allegedly broke out in a wet market of the city of Wuhan, the capital city of the Hubei Province, in Central China (30.60° N–114.05° E), where also the Wuhan Institute of Virology is located. The first case of hospital admission was reported on 12 December 2019 [1]. Since January 2020 the pandemic rapidly spread throughout the whole province of Hubei, in other regions of China and, further, began to spread all over the world. The COVID-19 pandemic is being monitored by the World Health Organization (WHO). (Web Page: https://www.who.int/emergencies/diseases/novel-coronavirus-2019, accessed on 15 April 2020. Coronavirus disease (COVID-19) Pandemic).

The high lethality rate of this virus requires the development of epidemic control strategies such as lock-down of the infected region and others [2], which, however, in time can negatively affect the economy of a society. Thus, it is a priority to forecast how the COVID-19 pandemic could geographically propagate and affect society for optimizing these strategies.

**Figure 1:**
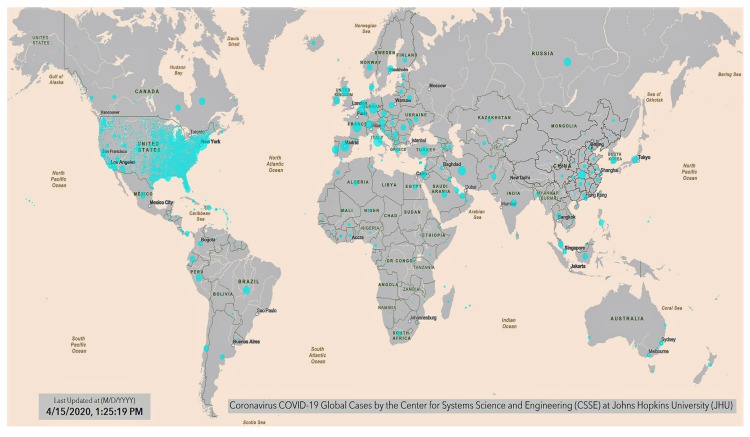
Geographical world distribution of the Coronavirus disease (COVID)-19 pandemic.

**Figure 2:**
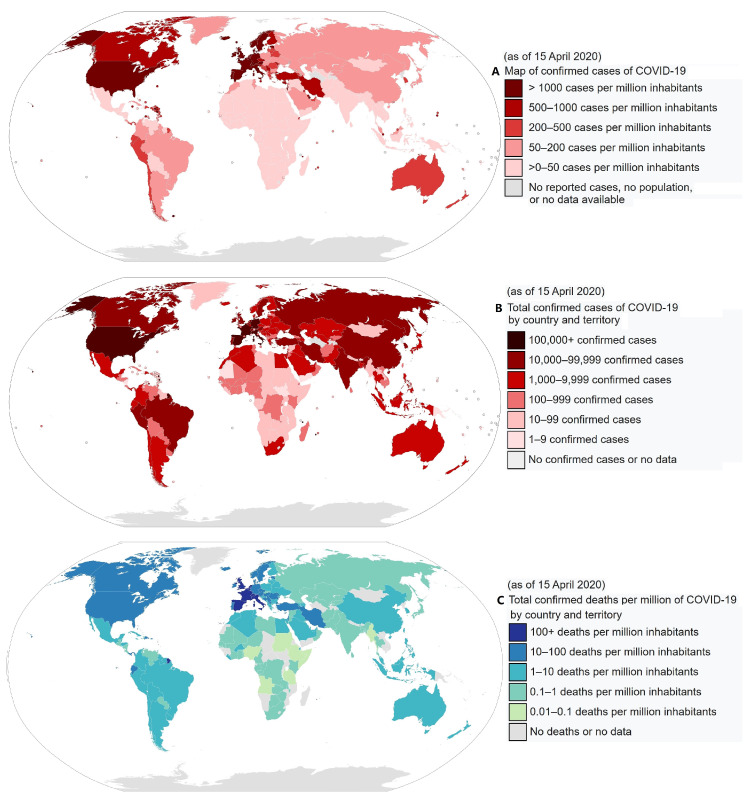
COVID-19 pandemic by country and territory: (**A**) confirmed cases per million inhabitants; (**B**) total confirmed cases by country and territory; (**C**) total confirmed deaths per million of COVID-19 by country and territory.

Data regarding the evolution COVID-19 Coronavirus Pandemic of confirmed cases and deaths by country, territory or conveyance are collected by numerous web-sites. Figure 1 shows the geographic distribution of the current COVID-19 global situation prepared by the Center for System Science and Engineering at John Hopkins University (as of 15 April 2020). (Source: https://gisanddata.maps.arcgis.com/apps/opsdashboard/index.html#/bda7594740fd40299423467b48e9ecf6, accessed on 15 April 2020.) Table 1 reports the first fifty-six countries affected by the COVID-19 pandemic ranked by total cases, total cases per million, total deaths and total deaths per million. (Web Page: https://www.worldometers.info/coronavirus/, accessed on 15 April 2020.) Figure 2 shows global geographical distributions of the three indices referring to the coronavirus pandemic by country and territory: [A] confirmed cases per million inhabitants; [B] total confirmed cases by country and territory; [C] total confirmed deaths per million of COVID-19 by country and territory. (Source: https://en.wikipedia.org/wiki/2019%E2%80%9320_coronavirus_pandemic_by_country_and_territory, accessed on 15 April 2020).

As of the date—15 April 2020—there are about 2 million people confirmed infected worldwide with about 130 thousand deaths versus only about 500,000 people recovered. China, where the infection first broke out in December 2019, officially had about 82,295 cases infected with 3342 total deaths, of which 3222 came from the province of Hubei alone. The Chinese authorities have declared that there have been very few cases of new infections since the first weeks of March and claim that since then in China the pandemic is under control (cf. Report of the WHO-China Joint Mission on Coronavirus Disease 2019. (Source: https://www.who.int/docs/default-source/coronaviruse/who-china-joint-mission-on-covid-19-final-report.pdf, accessed on 15 April 2020.) However, on 17 April, China acknowledged significant reporting errors regarding the total deaths, which were corrected from 3342 to 4632.

However, at the beginning of February, the pandemic began to show up outside of China spreading all over the world. South Korea was the first country severely affected by the pandemic (10,591 cases confirmed and 225 deaths), where since late March the pandemic has appeared mostly under control although in April the rate increased slightly. Later, many other countries were affected such as Iran, Turkey, several European countries (mostly Italy, Spain, France, Germany and the United Kingdom). In Europe, the situation is monitored also by the European Centre for Disease Prevention and Control. (Web page: https://www.ecdc.europa.eu/en/geographical-distribution-2019-ncov-cases, accessed on 15 April 2020.) Since the 15th of March the COVID-19 pandemic has been rapidly spreading in the United States of America, where, as of April 15, there have been more than 600,000 cases with more than 26,000 deaths.

**Figure 3:**
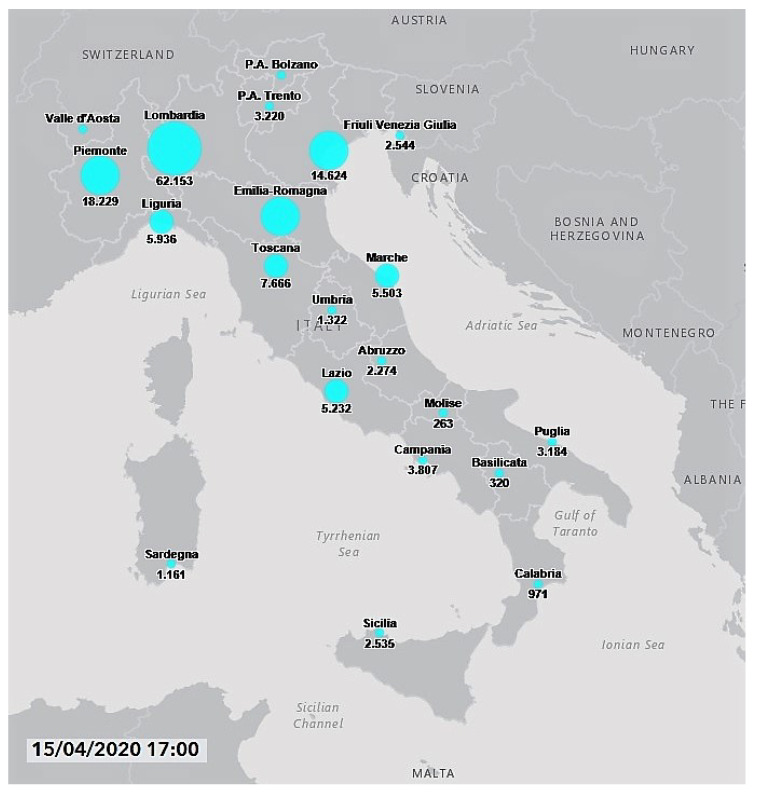
Geographical distribution of COVID-19 pandemic in Italy.

Italy has been one of the countries most severely affected by the COVID-19 induced pneumonia. (The situation in Italy: http://www.salute.gov.it/portale/nuovocoronavirus/homeNuovoCoronavirus.jsp?lingua=english, accessed on 15 April 2020.) The first three cases were identified on 20 February and, on 15 April there were more than 162 thousand confirmed infected people with more than 21 thousand deaths and only 38 thousand recoveries. The most affected Italian regions were the northern ones, those located in the Po Valley: Lombardia, 62,153 cases and 11,377 deaths; Emilia Romagna, 21,029 cases and 2788 deaths; Piemonte, 18,229 cases with 2015 deaths; Veneto, 14,624 cases and 940 deaths. (Source: http://www.salute.gov.it/imgs/C_17_notizie_4482_0_file.pdf, accessed on 15 April 2020.) In Lombardia, the most affected provinces were Milan (14,600 cases), Bergamo (10,472 cases) and Brescia (11,187). (Source: http://www.salute.gov.it/imgs/C_17_notizie_4482_1_file.pdf, accessed on 15 April 2020.) See Figure 3. (COVID-19 Italy situation: http://opendatadpc.maps.arcgis.com/apps/opsdashboard/index.html#/b0c68bce2cce478eaac82fe38d4138b1, accessed on 15 April 2020.)

Although the propagation of a pandemic and its health severity may have several causes including people’s network contacts, greater mobility, more concentrated demography, population age and air pollution as it usually happens in large and economically developed zones, Figure 2 suggests that the spread of the COVID-19 pandemic could also have a main geographical climatic preference. In fact, the pandemic appears to have spread quite fast in moderately cold places where the daily average temperature may have been roughly between 0 °C and 16 °C. On the other hand, up to 15 April, countries with warm climates (e.g., India, Central America, South America, Southern Asia, Africa and Oceania), as well as very cold countries (e.g., Canada, Russia, North-Eastern Europe) appear to have been less affected. It is also noticeable that even when the number of cases is relatively large relative to the population, the number of deaths is usually lower in warm countries, as shown in Figure 2C. Thus, it is reasonable to ask whether geographical regions within a specific climatic/weather zone could be more vulnerable to this epidemic. Indeed, several studies have established that influenza virus transmission and virulence depend also on meteorological conditions such as temperature, relative humidity and wind speed; besides, in the Northern Hemisphere, influenza is more widely spread during the winter seasons [3,4,5].

Influenza rates are normally seasonal [6] and, usually, these infections nearly disappear when the weather gets warm. Similar behavior has been observed for other SARS coronaviruses [7,8,9,10] that belong to the same Coronaviridae family of SARS-CoV-2 [11]. The finding has always been that these viruses spread mostly within given ranges of meteorological conditions. For example, Yuan et al. [12] determined that the SARS virus identified in November 2002 in Guangdong Province, China, presents a peak spread at a mean temperature of 16.9 °C (95% CI, 10.7 °C to 23.1 °C), with a mean relative humidity of 52.2% (95% CI, 33.0% to 71.4%) and wind speed of 2.8 m/s (95% CI, 2.0 to 3.6 m/s). Gaunt et al. [13] found that three coronaviruses showed “marked winter seasonality” and that they seemed to cause infections mainly between December and April. In a preliminary analysis, Bukhari and Jameel [14] noted that the novel COVID-19 pandemic has affected more seriously countries within a temperature range of 3 °C to 17 °C with absolute humidity between 3 g/m^3^ and 9 g/m^3^. Thus, also for the COVID-19 it has been proposed that a dry, moderate cold environment could be the most favorable state for the spreading of this virus [15].

The seasonal influence on the spread or slowdown of a pandemic induced by aerially transmitted viruses can have several biological, physical, solar-light mechanisms that involve both the virus survival and transmission in the air and the susceptibility of the host immune system [16]. Some of these results came from laboratory-experimental studies on how viral etiology and host susceptibility vary under different environmental conditions, while other findings are from epidemiological studies relating large-scale patterns to various climate signals and atmospheric conditions. In any case, even if viruses could survive at relatively high temperatures in lab experiments (indeed, they live in a human body at 37 °C or more) their ability to propagate from person to person and to overcome their immune system may strongly depend on seasonal weather conditions.

In this paper, geographical data describing the COVID-19 diffusion and lethality are correlated against weather-climatic conditions of various countries, their median population age and air pollution levels to determine the main possible factors affecting the pandemic. The study starts with a detailed comparison of the weather conditions from January to March 2020 between Wuhan and the Italian provinces of Milan, Brescia and Bergamo that were the first most affected regions. The striking similarity between the weather conditions of these two regions leads us to reason that there could be an optimal weather condition (dry and moderate cold weather) that could favor the spreading of the COVID-19 epidemic. This hypothesis is also supported by physical considerations related to the transmission in the air of viruses through respiratory droplets, as explained in the discussion section. The testing is then completed by analyzing the situation in the United States of America and finally of the entire world. Finally, the median population age of the countries and their air pollution levels are analyzed to evaluate their possible contribution to the pandemic. Based on this reasoning and the analysis results, it is suggested that weather-climatic conditions are the main factor regulating the development of the pandemic. Consequently, a set of optimized monthly isotherm world maps from January to December 2020 are proposed to forecast its course and timeline evolution by identifying the geographical regions that are likely to experience similar weather conditions as in Wuhan and Milan during the high peak of the COVID-19 infection. For each country and region, weather predictions could later be further convoluted with the median population age of the specific place since the COVID-19 affects mostly the elderly. The online supplementary files provide the same maps as Google-Earth-Pro interactive files and the full data files. (Appendix A).

## 2. COVID-19 and Weather-Climatic Conditions

Herein, the geographical diffusion and lethality of COVID-19 are analyzed against weather and climatic conditions.

### 2.1. COVID-19 in Wuhan (Hubei, China) versus Milan, Brescia and Bergamo (Northern Italian Provinces)

**Figure 4:**
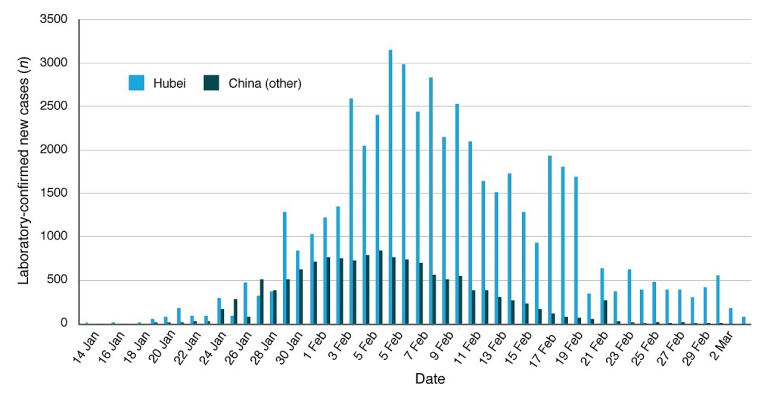
COVID-19 epidemic curve in Hubei and the rest of China. Data sourced from media reports, ProMED-Mail and WHO situation reports. (Adapted from [17]).

**Figure 5:**
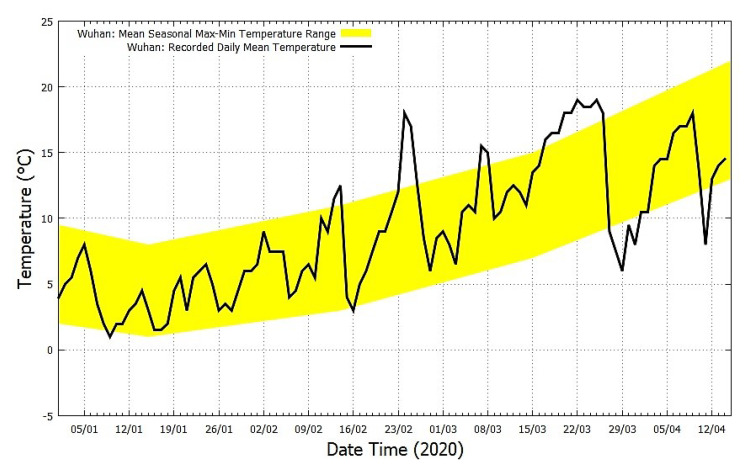
The daily mean temperature record of Wuhan (black) and its climatic temperature averages (yellow).

The COVID-19 epidemic curve in Hubei and the rest of China until the 3 March is shown in Figure 4 and its patterns are extensively commented in Macintyre [17]. It is observed that the epidemic peaked in the first week of February and the number of new infections rapidly decreased in March. Figure 5 shows the recorded mean daily temperature of Wuhan from 1 January 2020 to 14 April 2020 (black curve). (Source: https://www.accuweather.com/en/cn/wuhan/103847/current-weather/103847, accessed on 15 April 2020.) The figure also shows the mean seasonal Max-Min temperature range curve from Wuhan Airport, which is 23 km from Wuhan (yellow area). (Source: https://www.timeanddate.com/weather/china/wuhan/climate, accessed on 15 April 2020.) The temperature data show that in Wuhan the daily temperature roughly ranges between 1 °C and 8 °C in January, between 3 °C and 11 °C in February and 7 °C and 15 °C in March. During the period most affected by the COVID-19 infection (January and February), the temperature in the region roughly varied between 0 °C and 12 °C. The seasonal average relative humidity in Wuhan was on average around 70% and the average wind speed was around 10 km/h, which are the typical humidity and high-pressure conditions that characterize Continental China during winter when only the weak monsoon winds blow.

**Figure 6:**
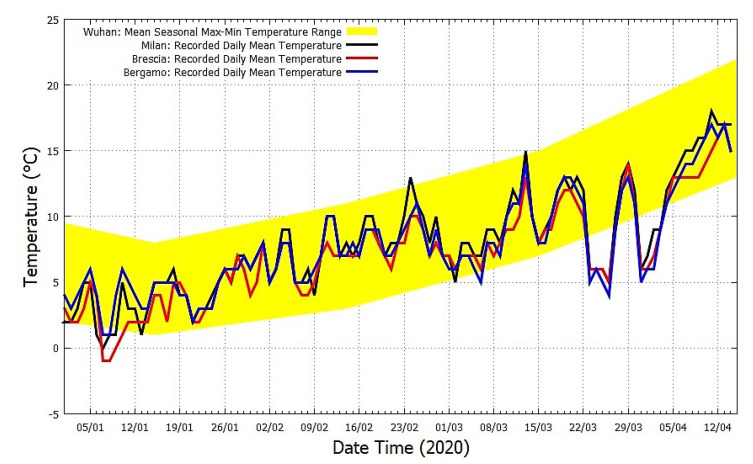
The daily mean temperature record of Milan, Brescia and Bergamo (black, blue and red) versus Wuhan’s climatic temperature averages (yellow).

Figure 6 compares the seasonal mean temperature of Wuhan (yellow area) with the recorded mean daily temperature records of the Italian cities of Milan, Brescia and Bergamo during the same months. (Source: https://www.ilmeteo.it/portale/archivio-meteo/Milano, https://www.ilmeteo.it/portale/archivio-meteo/Brescia, and https://www.ilmeteo.it/portale/archivio-meteo/Bergamo, accessed on 15 April 2020.) It shows an excellent correlation between the temperature records. This temperature correlation occurred mostly for January and February when Wuhan experienced a mean temperature equal to 4.1±3 °C and 8.4±3.6 °C, respectively. The Italian cities experienced a mean temperature equal to 3.8±2.0 °C and 8.1±2.1 °C, respectively, in Milan; 4.3±1.6 °C and 7.7±1.7 °C, respectively, in Bergamo; and 3.2±2.0 °C and 7.2±1.6 °C, respectively, in Brescia.

However, Figure 6 also highlights that the three Italian cities experienced a relatively cold March with a mean temperature equal to 9.5±2.8 °C in Milan, 8.8±2.9 °C in Bergamo and 8.7±2.4 °C in Brescia. In March, the daily mean temperature spanned between 4 °C and 15 °C. This temperature range fits well with the one measured in Wuhan in February where the mean temperature was equal to 8.4±3.6 °C and the daily mean temperature spanned between 3 °C and 18 °C. In March, Wuhan was significantly warmer: its mean temperature was equal to 12.9±4.1 °C and the daily mean temperature spanned between 6 °C and 19 °C.

The above results are summarized in Table 2 together with other meteorological indices such as monthly means of the relative humidity, wind speed and atmospheric pressure, which are also found to be comparable in the two locations. It is to be noted that the observed low relative humidity (from 61% to 85%), low-speed wind (from 6 km/h to 11 km/h), and high atmospheric pressure (from 1016 mbar to 1026 mbar) induce atmospheric stability facilitating the spreading of viruses. (Source for Wuhan: https://www.worldweatheronline.com/wuhan-weather-averages/hubei/cn.aspx, accessed on 15 April 2020.)

In January and February, the two locations shared strikingly similar weather conditions, but in March Wuhan got warmer fast while the Italian provinces experienced cold weather similar to that of Wuhan in February. These facts could explain why the COVID-19 pandemic spread equally fast in both regions, but the Italian regions were more severely affected. As Figure 6 shows, in Italy, the cold weather lasted longer with unusual cold weeks at the beginning and the end of March, favoring the pandemic spread.

Table 3 reports the COVID-19 death statistics as for 15 April 2020, the mean weather condition of March for each Italian region with their population densities. The table’s rationale is that most people who died were infected in March. The data indicate that the colder northern regions with temperature roughly ranging between 3 °C and 14 °C and with low-speed winds roughly ranging between 8 km/h and 12 km/h were those most affected by the pandemic. The number of deaths sharply decreases by moving toward the southern regions that were on average about 2 °C warmer. The latter regions also had stronger wind roughly ranging between 12 km/h and 15 km/h. Northern regions were also slightly dryer (RH = 66%) than the Southern ones (RH = 73%).

The Pearson correlation coefficients are calculated between the weather indices and the logarithm of the Deaths/1Mil record because the latter can be hypothesized to increase exponentially with environmental variables. In fact, it is expected that each person could infect or cause the death of a number α of people each day, which yields to a geometric sequence with an exponential growth in time as $N\left(t\right)={\left(1+\alpha \right)}^{t-1}$, where *t* is the number of days from the first infection. As a result that α is expected to be small with respect to 1 (for example, for *N*(50) = 1000 deaths per million people after 50 days of pandemic, α=0.15), we have that logN(t)∝(t−1)α, which is our model to be tested.

We obtain high significance correlation levels: $r_{T}=-0.49$ with $P_{20}\left(-1\leq r\leq r_{T}\right)=1.4\%$ for the temperature; $r_{H}=-0.37$ with $P_{20}\left(-1\leq r\leq r_{H}\right)=5.4\%$ for the humidity; and $r_{W}=-0.67$ with $P_{20}\left(-1\leq r\leq r_{W}\right)=0.1\%$ for the wind speed. Possible co-factors are discussed in Section 3.

The above considerations and statistical results suggest that weather temperatures roughly ranging between 4 °C and 12 °C together with weather conditions of low relative humidity and low-speed winds could be those that mostly favor the propagation of COVID-19 and/or aggravate the susceptibility of people to its secondary pneumonia.

### 2.2. COVID-19 in the United States of America

For 15 April 2020, the United States of America overtook Italy and China as the country with the highest number of confirmed COVID-19 cases with more than 600,000 cases (nearly 2000 cases per million people) and 26,000 deaths (nearly 86 deaths per million people). Indeed, the weather condition of the USA has been relatively similar to that of Western Europe in March and of that of Wuhan in February. The USA were chosen because the country is geographically vast and strongly interconnected, all states reacted nearly simultaneously to the pandemic and the available information is homogeneous and of high quality.

**Figure 7:**
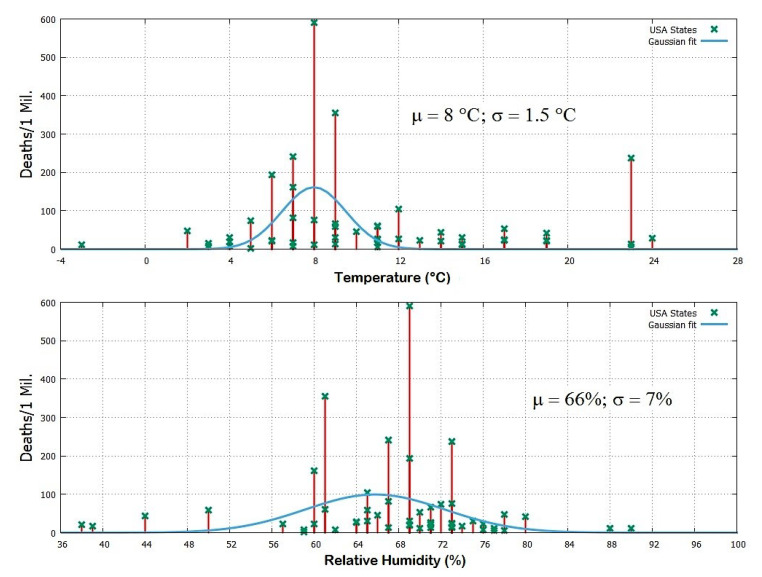
COVID-19 deaths per million people versus the mean temperature and mean relative humidity for March 2020 for the largest cities for each of the 51 states of the USA. See Table 4.

To test such a relation, mean temperature and relative humidity data were collected for March for each of the 51 states of the union using as reference their largest city and compared against the COVID-19 deaths per million people data for each State. (Web Pages: https://www.worldweatheronline.com, and https://www.worldometers.info/coronavirus/country/us/, accessed on 15 April 2020.) As above, the weather records of March were used because most of the people who have died by April 15 were very likely infected in March. The data are reported in Table 4 and plotted in Figure 7.

Table 4 and Figure 7 confirm that the CODIV-19 lethality has been worst in states with an average temperature ranging between 4 °C and 12 °C. In this temperature range, there are 30 states with an average of 82 deaths per million people with New York being the most affected state with 591 deaths per million people. There are other 21 colder or warmer states, and they had on average 34 deaths per million people.

The State of Louisiana appears anomalous. In fact, despite its 23 °C, it had 237 deaths per million people. Indeed, according to several news agencies, New Orleans has been a center of the coronavirus crisis likely because over 1.4 million people, coming from around the world, flocked to the city to celebrate a carnival for more than a month, culminating in the Mardi Gras celebration at the end of February. This event made probable that even a few persons could infect numerous people. (Sorce: https://www.nytimes.com/2020/04/13/us/coronavirus-new-orleans-mardi-gras.html, accessed on 15 April 2020.) By excluding the State of Louisiana from the above statistics, there were 20 States colder or warmer than the range from 4 °C to 12 °C which had on average 23 deaths per million people. This suggests that the COVID-19 lethality in the States with a mean temperature between 4 °C to 12 °C was on average nearly 4 times larger than in the other States: that is 80% versus 20%, respectively. Table 4 and Figure 7 also compare the mean relative humidity values for March for each of the 51 States of the USA. The records confirm that states with relative humidity levels between 60% and 75% were the most affected. Within this range 35 states are found with on average 81 deaths per million people. The other 16 states have on average 21 deaths per million people. This makes again a 80% versus 20%, respectively. Possible co-factors are discussed in Section 3.

Figure 7 fits the data with Gaussian functions of the type $f\left(x\right)=a\exp[-{\left(x-\mu \right)}^{2}/2\sigma^{2}]$ where *a* is the maximum value, μ = mean and σ = standard deviation. For the temperature data we get: a=162 deaths/1Mil; μ=1.5 °C; σ=1.5 °C. For the relative humidity data we get: a=100 deaths/1Mil; μ=66%; σ=7%.

The Pearson correlation coefficients are calculated between the logarithm of Cases/1Mil and Deaths/1Mil because they can be hypothesized to increase exponentially with the environmental variables versus the function f(y)=|x−μ|, where μ is the mean calculated above for the temperature and humidity: see also the explanation in Section 2.1.

Using Cases/1Mil, for the temperature it is found $r_{T}=-0.29$ with $P_{50}\left(-1\leq r\leq r_{T}\right)=2\%$, and for the humidity $r_{H}=-0.29$ with $P_{50}\left(-1\leq r\leq r_{H}\right)=2\%$. Using Deaths/1Mil, for the temperature it is found $r_{T}=-0.23$ with $P_{50}\left(-1\leq r\leq r_{T}\right)=5.4\%$, and for the humidity $r_{H}=-0.27$ with $P_{50}\left(-1\leq r\leq r_{H}\right)=2.9\%$.

If the State of Louisiana is excluded, for Cases/1Mil it is found $r_{T}=-0.40$ with $P_{49}\left(-1\leq r\leq r_{T}\right)=0.2\%$, and $r_{H}=-0.30$ with $P_{49}\left(-1\leq r\leq r_{H}\right)=1.8\%$. Using Deaths/1Mil, it is found $r_{T}=-0.34$ with $P_{49}\left(-1\leq r\leq r_{T}\right)=0.8\%$, and $r_{H}=-0.28$ with $P_{49}\left(-1\leq r\leq r_{H}\right)=2.6\%$.

The above considerations and statistical results suggest that weather temperatures roughly ranging between 4 °C and 12 °C together with weather conditions of low relative humidity between 59% and 73% could be those that mostly favor the propagation of COVID-19 and/or aggravate the susceptibility of people to its secondary pneumonia. Possible co-factors are discussed in Section 3.

### 2.3. World Statistics

Table 5 collects data regarding the COVID-19 pandemic situation for each continent as for 15 April 2020. Together with the patterns shown in Figure 1 and Figure 2, and the data reported in Table 1, the Western European countries and the United States of America, together with a few other countries with similar climatic conditions, have been most affected by the COVID-19 pandemic. Colder countries such as Canada and Asian Russia, as well as the warmer Central and Southern America, Africa, Southern Asia and Australia, have been scarcely affected. This is particularly evident by comparing the number of deaths per million people among the continents.

Mean monthly land temperature data from the Climatic Research Unit (CRU) Time-Series (TS) version 4.03, which are available from January 1901 to December 2018 [18], were collected for the 151 world countries having a population of at least one million people. (Sorce: https://crudata.uea.ac.uk/cru/data/hrg/cru_ts_4.03/, accessed on 15 April 2020.) As a result of the changing climate, monthly averages from 2000 to 2018 were used to determine their whether-climatic condition for March, when the pandemic spread around the world. These 151 countries were collected according to three climatic zones: (1) 13 cold countries with March mean temperatures lower than 2 °C; (2) 41 temperate countries with March mean temperature between 2 °C and 14 °C; and (3) 97 warm countries with March mean temperature higher than 14 °C.

Relative to the analysis result shown in Figure 7, the temperature range between 2 °C and 14 °C was used to allow a certain variability because on a world scale the pandemic did not start simultaneously in all countries and because the climatic averages can vary from the actual monthly values by about 2 °C. Sweden was added to the group (2) despite it belongs to the cold country group (1) because nearly all of its population lives in the warmer southern region. All data are reported in the Table provided in the Supplementary file.

**Figure 8:**
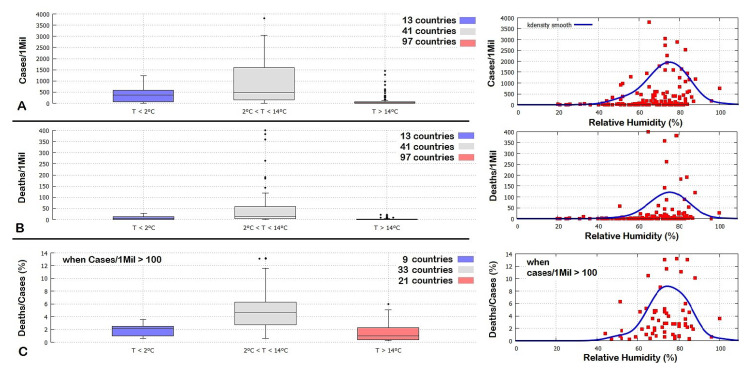
Left panels: Boxplots of country distribution in function of their March mean temperature, relative to the number of COVID-19 cases (**A**) and deaths (**B**) per million people, and the percent of deaths per cases (**C**). In (**C**) only the countries with more than 100 cases/1Mil were considered to reduce statistical volatility. See the supplementary file for the data. Right panels: equivalent scatterplots in function of the March mean relative humidity for the same countries (red) with the correspondent k density smooth function (blue).

Figure 8 shows boxplots that highlight the lower outliers, “minimum”, first quartile, median, third quartile, “maximum”, higher outliers for each of the three groups. They show the three temperature country groups in the function of their number of COVID-19 cases and deaths per million people and the percent of deaths per number of cases. In the latter case, only the countries with more than 100 cases/1Mil were considered. The boxplot values are reported in Table 6.

The boxplot analysis clearly shows that the countries with March mean temperatures ranging between 2 °C and 14 °C have been the most affected by the COVID-19 pandemic: their median of the number of cases per one million people is nearly 20 times larger than the median for the warm countries. On 97 warm countries, only 21 had a number of cases per one million people larger than 100. Furthermore, even if there are a few warm countries with a high number of COVID-19 cases per million people, their mortality rate has had a median 4.6 times lower than the countries belonging to the second group. Thus, the evidence is that getting infected in warm countries leads to a less severe clinical condition. The same finding, however, could have been also caused by the relatively low median population age of several warm countries such as the African ones, as discussed in the next section.

The Climatic Research Unit also provides monthly mean water vapor pressure data for the same countries and periods. We used the Tetens equation to calculate the saturation vapor pressure of water from the recorded temperature because of its ease of use and relative accuracy at temperatures within the normal ranges of natural weather conditions. (Web Site: https://en.wikipedia.org/wiki/Tetens_equation, accessed on 15 April 2020.) Then, we estimated the monthly average of the relative humidity (RH) values by calculating the ratio between vapor pressure and the saturation vapor pressure and, finally, the climatic average for each country and month using the data from 2000 to 2018, as done for the temperature. All RH data are collected in the supplementary files.

The data were analyzed in the following way. First, we divided the countries with more than 100 cases per million people (case A: 65 countries) from the others (case B: 88 countries). The data are shown in the right panels of Figure 8 which shows that, on average, countries with RH values roughly between 60% and 85% were the most affected by the COVID-19 pandemic with the rates falling significantly for RH values lower than 60%. We also divided the countries as done above, according to the same three temperature intervals. Set A has 9, 33 and 21 countries, respectively. Set B has 3, 8 and 76 countries, respectively. For the countries belonging to the Set A, we found an average of RH=73%±11% divided into the three temperature groups as: (1) RH=81%±13%; (2) RH=72%±7%; (3) RH=70%±14%. For the countries belonging to Set B, we found an average of RH=61%±17% divided into the three temperature groups as (1) RH=60%±15%; (2) RH=61%±11%; (3) RH=61%±18%. Thus, very dry climates appear to reduce the severity of the pandemic.

By repeating the Pearson correlation analysis between the logarithm of Cases/1Mil and Deaths/1Mil and the function f(y)=|y−μ| with μ=8 °C for the temperature and μ=72.5% °C for the humidity, it is found: for Cases/1Mil, $r_{T}=-0.55$ with $P_{151}\left(-1\leq r\leq r_{T}\right)\leq 0.01\%$, and $r_{H}=-0.23$ with $P_{151}\left(-1\leq r\leq r_{H}\right)=0.2\%$; for Deaths/1Mil, $r_{T}=-0.57$ with $P_{151}\left(-1\leq r\leq r_{T}\right)\leq 0.01\%$, and $r_{H}=-0.31$ with $P_{151}\left(-1\leq r\leq r_{H}\right)\leq 0.01\%$.

These results confirm those found for Italy and the USA, and further indicate that the COVID-19 pandemic diffusion and lethality rates are mostly related to weather-climatic conditions. The highest rates are found in places that in March had mean monthly temperatures roughly between 4 °C and 12 °C and with relative humidity roughly between 60% and 85%. These results are consistent with the analysis of the previous sections. Possible co-factors are discussed in Section 3.

## 3. COVID-19: Possible Co-Factors

This section discusses possible co-factors that could contribute to the diffusion and severity of COVID-19. These are the local median population age and air pollution quality.

### 3.1. COVID-19 and Countries’s Median Population Age

Data collected as to date (16 April 2020), although still incomplete, suggest a demographic influence on the COVID-19 pandemic. (Sorce: https://www.worldometers.info/coronavirus/coronavirus-age-sex-demographics/, accessed on 16 April 2020.) For example, preliminary works from China suggest that the probability of dying if infected by the virus is larger for males than females, it is larger in the presence of pre-existing health conditions, and it is significantly larger for the elderly than for younger people. (Source: The Novel Coronavirus Pneumonia Emergency Response Epidemiology Team: The Epidemiological Characteristics of an Outbreak of 2019 Novel Coronavirus Diseases (COVID-19)—China CCDC, 2020, 2(8): 113–122. http://weekly.chinacdc.cn/en/article/id/e53946e2-c6c4-41e9-9a9b-fea8db1a8f51.) These results seem to be confirmed worldwide. For example, data provided by New York City Health as of 14 April 2020, show that among the reported 6839 COVID-19 deaths, 2530 (37%) were females, 4095 (60%) were males and 215 (3%) were unknown. The share by age was as follows: 0–17 years old, 3 cases (0.04%); 18–44 years old, 309 cases (4.5%); 45–64 years old, 1581 cases (23.1%); 65–74 years old, 1683 cases (24.6%); 75+ years old, 3263 cases (47.7%). (Source: https://www1.nyc.gov/assets/doh/downloads/pdf/imm/covid-19-daily-data-summary-deaths-04152020-1.pdf, Accessed on 15 April 2020.) This evidence suggests that demographic statistics could significantly affect the COVID-19 pandemic and its consequences.

Regarding the situation for Italy, Table 3 also reports the regional population density and mean population age. (Source: http://www.comuni-italiani.it/statistiche/eta.html, accessed on 15 April 2020.) The population density does not appear to be significantly correlated to the pandemic rates as there are regions with low or high density both in the upper and lower part of the table: $r_{PD}=0.12$ with $P_{20}\left(r_{PD}\leq r\leq 1\right)=31\%$. On the contrary, a more significant correlation is found between COVID-19 and the mean population age: $r_{MA}=0.34$ with $P_{20}\left(r_{MA}\leq r\leq 1\right)=7.1\%$. In fact, regions with a higher percentage of elderly are usually more affected by the pandemic, but there are important exceptions that weaken the correlation pattern. For example, Lombardia was by far the most affected region (Deaths/Cases = 18.3%) despite its relatively low mean population age (44.3 years), while thirteen regions have a larger percentage of elderly, including the province of Basilicata which had the lowest Deaths/Cases value (6.6%). Thus, the population age is co-factor, but it does not seem to be the main variable determining the pandemic diffusion.

Regarding the situation for the USA, Table 4 also reports the median population age for each State. (Source: http://www.statsamerica.org/sip/rank_list.aspx?rank_label=pop46&ct=S09. Median Age in 2018. Accessed on 15 April 2020.) The comparison of these data versus the COVID-19 ones shows only a modest and, probably, not significant correlation ranging between $r_{MA}=0.10$ with $P_{50}\left(r_{MA}\leq r\leq 1\right)=24\%$ and $r_{MA}=0.17$ (excluding the State of Louisiana) with $P_{49}\left(r_{MA}\leq r\leq 1\right)=12\%$ according to the adopted record. In fact, the USA median population age varies between 31 and 44.9 years with an average of 38.5 years. By considering the first twenty States most affected by COVID-19, their mean median population age is 38.7 years for Cases/1Mil, 38.8 years for Deaths/1Mil and 38.6 years for Deaths/Cases; just above the national mean. The State of New York has a median population age of 39 years and has been the most affected one, despite there are nineteen States with a higher percentage of elderly including the cold Montana (3 °C) and the warm Hawaii (23 °C) that were among the States less affected by COVID-19.

**Figure 9:**
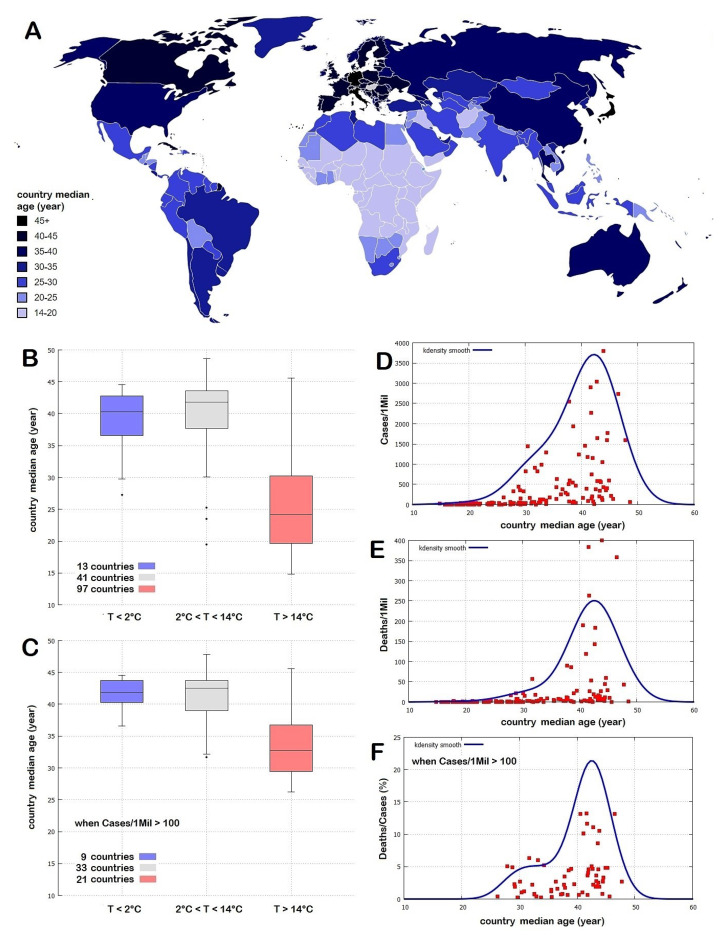
(**A**) World distribution of countries by mean population age. (**B**,**C**) distributions of countries by mean population age versus the climatic temperature zones depicted in Figure 8. (**D**–**F**) COVID-19 pandemic versus mean population of the countries.

Regarding the situation for the entire world, Figure 9A shows the world map of the countries relative to their median population age and it appears that the most affected countries—e.g., the European and the Northern American ones—could be those with the highest population age and, therefore, with the highest percentage of the elderly.

To test a possible demographic age effect and compare it against the weather patterns analyzed above, we show in Figure 9B,C boxplots of the country median age against the same sets of countries divided into weather temperature zones used in Figure 8.

The analysis shows that, while a large number of warm countries (*T* > 14 °C in March) also have a low median age, both the cold countries (*T* < 2 °C in March) and the temperate countries (2 °C < *T* < 14 °C) have a compatible median population age. Figure 9D,E show that the world countries distributions per Cases/1Mil and Deaths/1Mil per country median age peak for countries with a median age of around 40–45 years, but in the former case the distribution is significantly wider than in the latter case. In fact, Figure 9F shows that the distribution of Deaths/Cases per country median age appears to be made of two superimposed distributions: a uniform one per median ages between 27 and 47 years and values up to 5%, plus a set of seven countries (Italy, Spain, The Netherlands, France, Belgium, Sweden, the United Kingdom) with Deaths/Cases rates larger than 10%. These seven countries belong to the same weather-climatic region.

By repeating the Pearson correlation analysis between the logarithm of Cases/1Mil and Deaths/1Mil and the median age of the country population, it is found: for Cases/1Mil, $r_{MA}=0.8$ with $P_{151}\left(r_{MA}\leq r\leq 1\right)\leq 0.01\%$ and for Deaths/1Mil, $r_{MA}=0.7$ with $P_{151}\left(r_{MA}\leq r\leq 1\right)\leq 0.01\%$. However, these results are incompatible with those found in Italy and in the USA and suggest a coincidental finding induced by the large number of warm countries that also have a low median population age. In fact, the correlation does not hold by restricting the sample.

For example, Table 7 indicates that among the first ten countries with the highest median population age (between 44.4 and 48.6 years) only Italy had a significant death rate (Deaths/1Mil = 358) while the other nine countries had a significant lower lethality rate ranging between Hong Kong (Deaths/1Mil = 0.5) and Portugal (Deaths/1Mil = 59). Among the first five countries with the highest median population age (between 45.3 and 48.6 years), Japan and Germany have a larger median population age while Hong Kong and Greece have a lower median population age than Italy. The relatively low death rate of these four countries relative to that observed in Italy is better explained by their different climatic-weather conditions because, in March, Japan and Germany are significantly colder, while Greece and Hong Kong are significantly warmer than Italy by several Celsius degrees. The complete table for all 151 countries with more than one million people is found in the Supplementary Materials.

These results suggest that although the median population age of a country is an important co-factor for the COVID-19 diffusion and lethality rates, such a parameter is not as important as the climatic-weather ones. The same conclusion can be derived from the time evolution of the pandemic that, for example, in Europe from February to May, evolved from the warm South-Ovest region (Spain and Italy) toward colder Nord-Eastern region, with the cold Eastern-European countries that were the least affected despite their high median population age.

### 3.2. COVID-19 and Air Pollution

Atmospheric stability related to high atmospheric pressure and low-speed winds also cause a higher concentration of air pollutants [19] that could carry viruses. Air pollution increases the risk of respiratory diseases by reducing lung function making people living in particularly polluted areas more susceptible to asthma, respiratory infections, lung cancer and other diseases [20]. Air pollution has been explicitly considered to be a co-factor in the extremely high level of SARS-CoV-2 lethality in Northern Italy [21].

**Figure 10:**
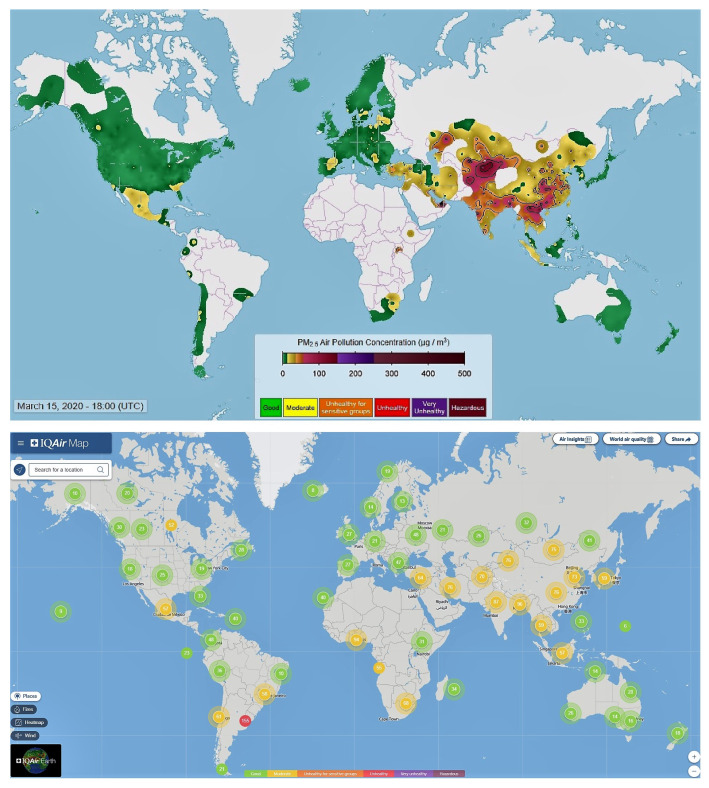
Top: near real-time information on particulate matter air pollution less than 2.5 microns in diameter (PM2.5). The image refers to 15 March 2020, at 18:00 UTC. Bottom: a typical World Air Quality Index (AQI) map from IQAir.

However, as Figure 10 shows, air quality real-time world-maps provided, for example, by Berkeley Earth or by IQAir suggest that the air in regions such as China, India and South-Eastern Asia is significantly more polluted than in the European and Northern American countries. (Web pages: http://berkeleyearth.org/air-quality-real-time-map/, and https://www.iqair.com/, accessed on 15 April 2020.) In general, the world pandemic patterns shown in Figure 1 and Figure 2 and the world pollution patterns shown in Figure 10 are very poorly correlated. On the considered country scale, the correlation appears even negative as if air pollution could reduce the COVID-19 diffusion and lethality, which would be counter-intuitive based on the known negative effects of continued air pollution exposure on human health.

In fact, from Table 5, Europe had 1292 cases and 118 deaths per million people while Asia had 72 cases and 2.6 deaths per million people (note that Asian Russia does not count much because it is scarcely populated). Moreover, by comparing the Country Air Quality Index by IQAir (Table 8) against the logarithm of the country Cases/1Mil and Deaths/1Mil, we obtain the following strong negative Pearson correlation coefficients: for Cases/1Mil, $r_{IQR}=-0.48$ ($P_{90}\left(r_{IQR}\leq r\leq 1\right)=100\%$) and for Deaths/1Mil, $r_{IQR}=-0.46$ ($P_{84}\left(r_{MA}\leq r\leq 1\right)\leq 100\%$).

In Italy it is possible to highlight the emblematic case of the small Republic of San Marino located on the hills between the regions of Emilia-Romagna and Marche. San Marino has been the country in the world with the highest number of infections per million people (Cases/1Mil = 11,632) despite its cleaner air, while the nearby Italian province of Rimini was significantly less affected (Cases/1Mil = 5214). It is possible that this happened because, despite their higher air pollution levels, the main cities of this province (Rimini, Cattolica, Riccione) are located nearby the coast and are a few Celsius degrees warmer than San Marino, whose temperature in March could have been close to the critical temperature of 8 °C.

Thus, the above results suggest either that air pollution mitigates the CONVID-19 pandemic (which appears unlikely), or the found negative correlation is accidental because the phenomenon is driven by other major factors such as weather-climatic conditions.

In fact, the main viral carriers are not aerosols, particulates (PM10 or PM2.5) or other typical pollutants but, rather, water droplets exhaled by infected people when they blow their nose, cough, sneeze, or just breathe [22]. The physical characteristics and dynamics of such water droplets in the air strongly depend on the weather conditions of the place such as temperature, relative humidity and wind speed.

In conclusion, the data show a relatively low COVID-19 diffusion and mortality rates in the warm but polluted Asian countries in contrast to the high mortality levels of the colder but less polluted European countries and the United States of America (see Figure 2). This suggests that weather, not air pollution is the main cause for the diffusion and lethality of the COVID-19 pandemic, at least at the considered synoptic scale.

## 4. Monthly Isotherm World Maps

**Figure 11:**
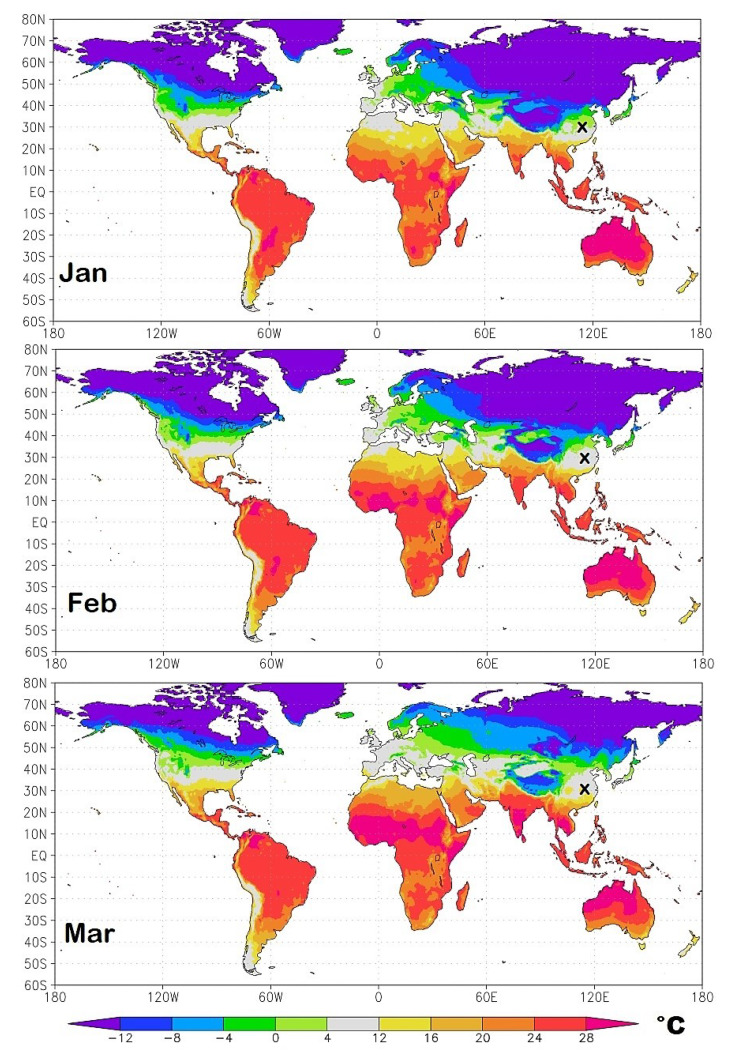
Winter isotherm world maps from January to March. The small x over China indicates the position of Wuhan. The light-gray zone represents the modeled most exposed regions to the COVID-19 pandemic.

**Figure 12:**
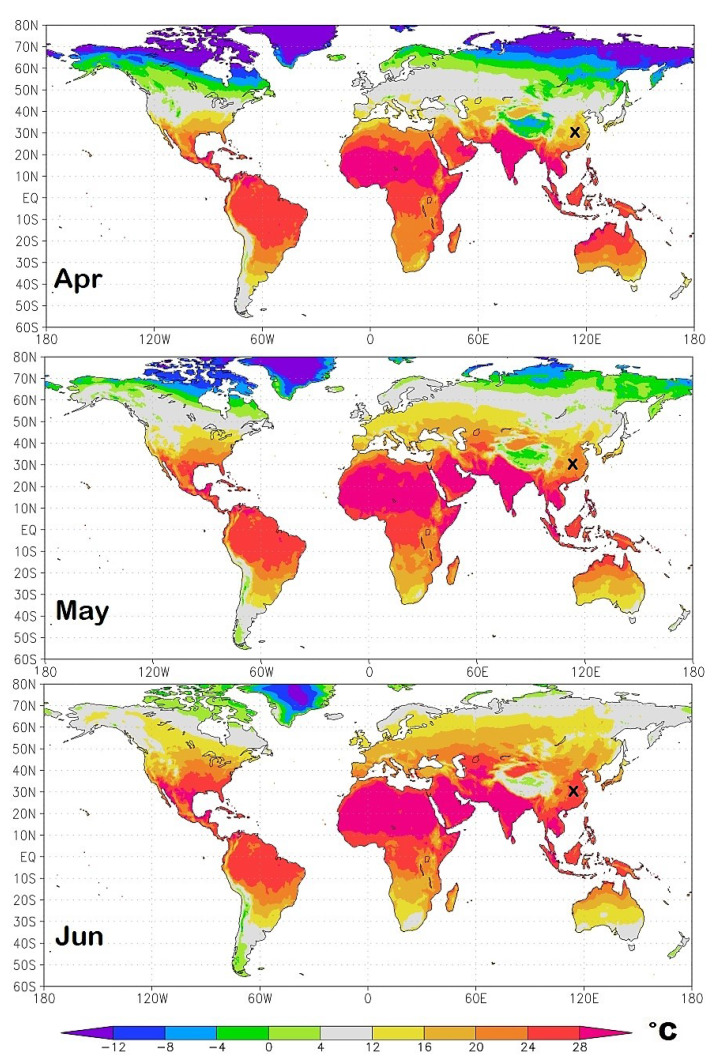
Spring isotherm world maps from April to June. The small x over China indicates the position of Wuhan. The light-gray zone represents the modeled most exposed regions to the COVID-19 pandemic.

**Figure 13:**
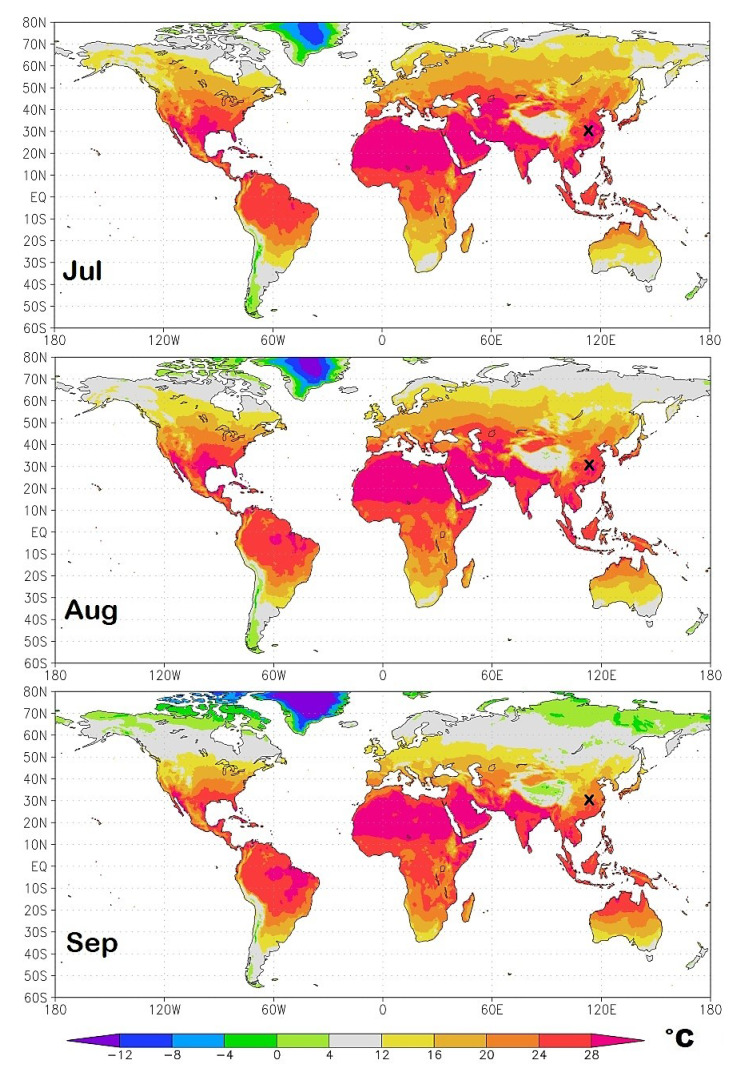
Summer isotherm world maps from July to September. The small x over China indicates the position of Wuhan. The light-gray zone represents the modeled most exposed regions to the COVID-19 pandemic.

**Figure 14:**
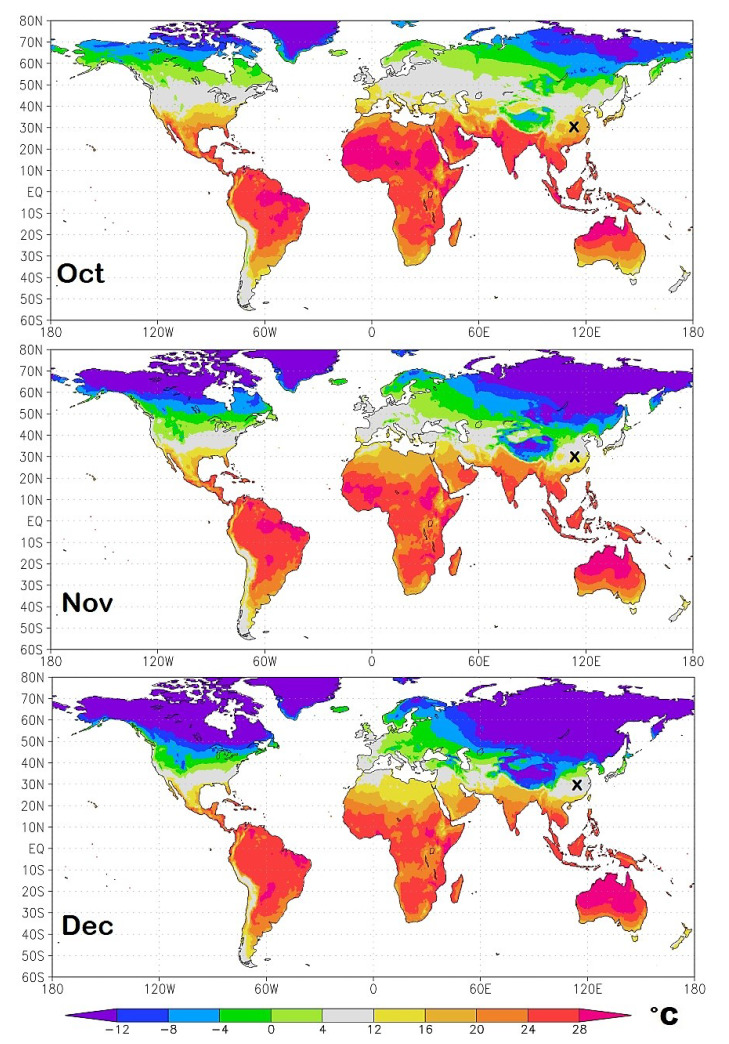
Autumn isotherm world maps from October to December. The small x over China indicates the position of Wuhan. The light-gray zone represents the modeled most exposed regions to the COVID-19 pandemic.

As a result of the seasonal cycle, the geographical regions most exposed to the initial infection continuously change in time. To identify them I propose in Figure 11, Figure 12, Figure 13 and Figure 14 isotherm world maps for each month of the year from January to December. For a correct interpretation of the diagrams, note that the disease manifests itself about two-three weeks after the infection.

The temperature data used are from the Climatic Research Unit (CRU) Time-Series (TS) version 4.03 of high-resolution 0.5° × 0.5° gridded data of month-by-month variation in land temperature, which are available from January 1901 to December 2018 [18]. As a result of the changing climate, the depicted diagrams are based on monthly averages from 2000 to 2018.

To better highlight the geographical zone of interest, the chosen colors cover the following bands: light-green (0 °C–4.0 °C); light-gray (4.0 °C–12 °C), the likely most affected zone estimated above; and light-yellow (12 °C–16 °C). The colder and warmer zones are colored differently, as indicated in the legend. The figures also show a small “x” over China, which indicates the approximate position of Wuhan. Data and graphical tools to reproduce the graphs are available also on KNMI Climate Explorer. (Source: https://climexp.knmi.nl/start.cgi, accessed on 15 April 2020.)

For the winter months of January, February and March, the isotherm maps depicted in Figure 11 show a geographical correlation with the COVID-19 pandemic patterns by country and territory shown in Figure 1 and Figure 2. The light-gray area is the most affected, while the colored areas, both colder or warmer, are those that have currently experienced a less severe pandemic.

- January—Wuhan gradually turns from the light-green zone to the light-gray zone as this region gets warmer.
- February—when the pandemic affected the region most severely, Wuhan is found in the middle of the light-gray zone. The light-gray zone covers the region spanning from Iran to Italy, Spain, and partially covers Southern-East France and North Algeria. In these countries, the epidemic has been observed to spread fast.
- March—Wuhan gets warmer fast as it enters the light-yellow zone and its infection rate drops. In the meantime, the light-gray zone moves slightly toward North-East involving all Western European countries including Germany and the United Kingdom. The northern region of Italy where Milan, Brescia and Bergamo are located—just south of the Alps that are recognized in the figure by the dark green arc above Italy—is in the middle of the light-gray zone as Wuhan was in February. The light-gray zone also covers most of the United State of America. Indeed, in March 2020, all countries mentioned above have experienced a significant acceleration of the pandemic.
- April—the light-gray zone moves toward North-Eastern Europe and Russia and, in North America, toward Canada. In the United States, the east region gets warm fast and enters the light- and dark-yellow zone, while most of the west side remains in the light-gray zone. In the meantime, Chile and Southern Argentina enter the light-gray zone.
- May—the light-gray zone moves toward latitudes larger than 50° N mostly in the Scandinavian countries, Russia and Canada, as well Argentina, Chile and New Zealand.
- June—the only light-gray regions are those above 60° N latitude, the Tibet, part of Central Argentina and minor regions of Southern Australia and South Africa.
- July—the patterns are similar to those of June, although the Northern Hemisphere continues to get warm.

From August to December (Figure 13 and Figure 14), the seasonal movement of the temperature patterns reverts: August is similar to June; September is similar to May; October is similar to April; November is similar to March; and December is similar to February.

Similar diagrams could be obtained for water vapor pressure or relative humidity. However, they would not add much information because these variables are partially related to the air temperature patterns. In general, Figure 8 showed that very dry climates (RH < 60%) appear to reduce the spread of SARS-CoV-2.

## 5. Discussion and Conclusions

Respiratory virus infection rate is usually seasonal [7,8,9,10]. This applies also to the coronaviridae family of COVID-19 [11]. In general, there are likely several biological, physical and sunlight mechanisms that can seasonally influence the survival and transmission of viruses in the air, as well as the susceptibility of the host immune system [16]. The weather conditions that facilitate this type of diseases include moderate cold and dry weather, high pressure, low-speed wind and modest rain, as it happened from January to March 2020 both in Wuhan and in Northern Italy. Similar weather conditions occurred in other countries where the pandemic has been significant, for example in Western Europe and the United States of America.

The COVID-19 infection primary transmission route from person to person is through contact with respiratory water droplets exhaled by infected persons. Contacts with surfaces contaminated by secretions or oral nose pharyngeal emissions could be a secondary transmission route. In fact, a person can also catch the virus if he or she touches a surface or object that has the virus on it and then touches his or her mouth, nose, or eyes.

Exhaled water droplets floating in the air could be as small as 0.5 to 10 micron and carry around coronaviruses, which have a size of about 120 nm. Such droplets are generated when, for example, people blow their nose, cough or sneeze, or just breathe. The warm and humid lungs add moisture to the breath. Thus, when people exhale on a cold day, the water vapor contained in their breath partially condenses as the air cools down. Therefore, when the air temperature is low, but not freezing, and the relative humidity is neither too low nor too high, as it happens in the winter in the most affected regions, water-based small droplets, which could carry viruses, more easily form and remain floating in the air for a longer time because they do not evaporate easily nor fall fast on the ground by further capturing additional water vapor. Thus, viruses could survive floating in the air protected by a small respiratory droplet long enough to infect somebody else who could pass by and then breathe in those droplets.

High temperature and dry air prevent the formation of such small condensation droplets or make them evaporate fast exposing the virus directly to the air which should induce a rapid destruction of its capsid. Larger droplets emitted with the cough fall rapidly to the ground and evaporate fast if the air is warm or very dry inducing again a rapid death of the virus. Freezing weather favors the formation of large droplets that fall fast on the ground. Rain facilitates the removal of virus-carrying droplets and virions because it captures them in the air and makes them fall on the ground. High atmospheric relative humidity would also facilitate the fall of such droplets by preventing them to become sufficiently small to float in the air for a time long enough to reach and, therefore, contaminate far distances. High atmospheric pressure reduces the wind speed, and virus-carrying droplet density could increase in the urban area. Northern Hemisphere winters also have fewer hours of sunlight and UV exposure that have a sterilizing effect [23]. In addition, cold weather usually increases the susceptibility of people to virus attacks, whereas summer warmer temperatures, more abundant UVB sunlight, and vitamin-rich food strengthen the immune system.

Cold weather also forces people to gather more likely in closed warm environments. Here it is easier to get infected because of poorer air exchange. In fact, in indoor environments—such as apartments, offices, shops, schools, restaurants, cruise ships, etc.—although the water of the infected droplets could evaporate faster exposing the virus to an inhospitable environment, the air concentration of floating virions, which could still remain active for a while, could increase so much to easily infect other people [22]. Indoor environments are also not protected nor sterilized by UV solar radiation: a fact that prolongs the life of the virus in such environments.

Thus, there are numerous direct and indirect mechanisms supporting the hypothesis that seasonal weather conditions affect the diffusion of SARS-CoV-2, as it has been observed for many other respiratory viral infections.

In any case, the virus by itself can survive also high temperatures, as it lives within human bodies and, therefore, people could get infected also in warm places, as it has been observed worldwide. This evidence, however, does not contradict the fact that the COVID-19 pandemic has been worst and developed more rapidly in specific countries which, during winter 2020, shared a common weather condition. The few observed anomalous cases could have simple explanations.

For example, as of the date (15 April 2020), the warm State of Louisiana in the United States of America counted 237 deaths per million people and was the fourth most affected state of the USA. However, this apparent anomaly was likely due to its famous carnival festivals that attracted into the city historic center 1.4 million people from all around the world, making the city the main infection hub. Similarly, the coronavirus outbreak in the city of Daegu in South Korea was induced by a very large service gathering by the Shincheonji religious group in a closed space. (Web Page: https://edition.cnn.com/2020/02/26/asia/shincheonji-south-korea-hnk-intl/index.html, accessed on 15 April 2020.)

We also checked whether air pollution could have been a relevant co-factor for the SARS-CoV-2 lethality in some countries. Our analysis suggests a poor correlation because heavily polluted countries such as India and the South-Eastern Asian countries (including also Southern China, Hong Kong, and Taiwan) have been modestly affected by the pandemic. For example, as of the date (15 April 2020), despite its close connections with China, Hong Kong (7,392,000 people by 2017) had about one thousand cases with only four deaths. Note that the COVID-19 started earlier in Hong Kong than in Italy because on 02/15/2020 in Hong Kong there were 56 cases while in Italy there were 3 cases. (Source: https://www.worldometers.info/coronavirus/, accessed on 15 April 2020.) The warm and dry Qatar, Bahrein and the United Arab Emirates (UAE) with their crowded and very polluted capitals (Doha, Manama and Dubai: Source: https://www.thenational.ae/uae/world-environment-day-middle-eastern-cities-choked-by-air-pollution-1.870540, accessed on 15 April 2020) had a number of COVID-19 cases—1288, 982 and 542 cases/1Mil, respectively—probably due to their international connections, but only 2, 4 and 3 deaths per million people, respectively. This is a mortality rate per infected people nearly 50 times lower than that observed in Italy and other Western European countries. By contrast, the small Republic of San Marino located on hills in Italy between the regions of Emilia-Romagna and Marche was much more affected by the COVID-19 pandemic than the nearby cities despite its cleaner air. This could have happened because San Marino is a few degree colder than the nearby warm cities located in the valley and on the coast.

Indeed, as of the date (15 April 2020), on 151 countries of the world with a population larger than one million people, among the 80 countries that had at least 50 cases/1Mil, those with a mortality rate larger than 10% were seven and from Western Europe: Belgium, Italy, and the United Kingdom, 13%; France, 12%; Netherlands, 11%; Spain and Sweden, 10%. On the contrary, the last seven countries were all from warm zones: the United Arab Emirates (UAE), 0.6%; Oman, Bahrain, Hong Kong, 0.4%; Singapore 0.3%; Kuwait, 0.2%; and Qatar, 0.15%. Note that the latter seven countries are internationally well interconnected, with high densely populated cities and medical technology not superior to that of the European countries. Thus, the different lethality rates, by a factor of 20 and above, observed between the two groups are likely due to their very different winter climatic conditions, with the European countries significantly colder than the latter.

Among the other 72 countries with less than 50 cases per million people, none is from Europe. There are 43 warm African countries except for the colder Marocco (Cases/1Mil = 55; Deaths/1Mil = 3, and Deaths/Cases = 5.5%) and Tunisia (Cases/1Mil = 66; Deaths/1Mil = 3, and Deaths/Cases = 4.5%), with the addition of the Mauritius islands (Cases/1Mil = 255, Deaths/1Mil = 7, and Deaths/Cases = 2.7%; note that these islands are a popular touristic place which could explain the relatively high infection rate). Large but warm countries such as Indonesia and India had just 19 and 9 cases per million people, respectively.

Thus, the relatively low COVID-19 diffusion and mortality rates, and the low ratio between mortality and infection rates observed in warm countries (see Figure 11) relative to the high mortality levels of colder countries such as those in Europe and the United States of America indicate that weather, not air pollution is the main factor for the diffusion and lethality of the COVID-19 pandemic. A correlation between the number of COVID-19 cases and higher polluted areas, such as large cities, could be accidental, and simply due to the higher population density of these places where it happens that more air pollution is also produced. In general, air pollution is not expected to be a major carrier of the coronavirus since the virus is mostly diffused in the air by human breath. The question about whether people living habitually in air polluted areas could be more sensitive to the infection because of a weakened immune system is a different topic, but still, pollution appears to only play a secondary role relative to weather conditions.

Finally, we checked a possible influence on the COVID-19 pandemic of the median population age of the place. The analysis was carried by comparing the regions of Italy, the United States of America, and the countries of the world. We did find a correlation with the COVID-19 but, by considering all local and global evidences, it appears weak relative to the one observed with the weather-climatic patterns. Thus, we conclude that places with a higher median population age could be disadvantaged, but still, the severity of the pandemic is driven mostly by the weather-climatic conditions that regulate both the diffusion of the virus through the air and the susceptibility of the immune system of the people to viral attacks. An additional detailed analysis may need to compare the weather records using age-standardized COVID-19 records divided by age categories, but this operation requires additional detailed data for each countries and regions. Whether this additional analysis could improve the reliability of the results herein presented is left to further research.

With regards to the mortality rates, a role is also played by the different quality of the health system of each country and region, but this analysis goes beyond the purpose of this work. For example, there have been reports of a large number of infections occurred within elderly care housing centers and hospitals, which had to and should be prevented. E.g., Wang et al. [24] found that in a single-center case series of 138 hospitalized patients with confirmed NCIP in Wuhan, China, presumed hospital-related transmission of 2019-nCoV was suspected in 41% of patients, 26% of patients received ICU care and mortality was 4.3%.

Based on the above considerations, this work explored the possible link between COVID-19 pandemic and weather conditions. We showed that the region of Wuhan in the Hubei Province, in Central China, and the Italian provinces of Milan, Brescia and Bergamo—which to date have been the most affected by the COVID-19 pandemic—presented a striking similarity in weather conditions between January and March. In particular, the weather condition in Wuhan in late January and February—when the COVID-19 infection affected that region most severely—was nearly identical to the weather conditions between February and March experienced in the Italian northern provinces. The same correlation was confirmed by a detailed analysis of the relation between weather conditions and the pandemic situation in the United States of America. These findings suggest that weather temperatures between 4 °C and 12 °C together with low humidity values between 60% and 80% and low-speed winds (about 10 km/h) could be those that mostly favor the spread of COVID-19 and/or aggravate the susceptibility of the population to its secondary pneumonia.

We used this result to create a specific isotherm world map for each month from January to December to highlight the timing and the position of the world regions that could be most affected by the pandemic in the upcoming months. To date—15 April 2020—the model appears to have well described the pandemic evolution as, for example, it has well predicted the pandemic strong development from February to the end of March in Iran, Italy, Spain, France, Germany, the United Kingdom and the United States of America, in order of time. Thus, it is possible that in the absence of adequate prevention policies, as the weather gets warm, the pandemic is likely to move following the seasonal temperature cycle and could migrate toward northern regions of the Northern Hemisphere while weakening in the southern ones such as China and Italy. The Southern Hemisphere appears to be more protected because most of its land, except for a few regions, is always sufficiently warm throughout the year. In addition, also the relatively low median population age of several warm countries, such as the African ones, could contribute to mitigating the effect of the pandemic in those countries.

Furthermore, the weather model suggests that in the Northern Hemisphere there may be a possible second wave of infections in the autumn following the return of the cold season. In general, the pandemic could return to the middle latitude regions (roughly 30°–60° N and 30°–60° S) with a 6-month cycle and in the other regions with an annual cycle. Although the transmission of COVID-19 should go down as weather temperature goes up, the virus may not disappear completely. The infection rate could simply slow down, as suggested by the evidence that people get infected also in warm regions, although in these places the percentage of deaths per million people appears to be significantly lower than in the cold weather regions (Figure 2). Thus, although the optimized isotherm maps proposed in the present work could be useful to optimize the timing of the required COVID-19 epidemic control policies that each country needs to implement, people and governments should be warned against lowering their guard.

## Figures and Tables

**Table 1:** Statistics of the first fifty-six countries (with more than one million people) most affected by the COVID-19 pandemic according to four metrics as of 15 April 2020. (Eu) Europe; (NA) North America; (SA) South America; (As) Asia; (Af) Africa; (AO) Australia-Oceania. * For countries with Cases/1Mil > 100. ** On 17 April 2020, China corrected the record and reported 82,692 cases and 4632 total deaths. The complete table is found in the Supplementary Materials.

	**Country**	**Total Cases**		**Country**	**Cases/1Mil**		**Country**	**Total Deaths**		**Country**	**Deaths/1Mil**		* Country	**Deaths/Cases**
(NA)	USA	641,410	(Eu)	Spain	3799	(NA)	USA	28,394	(Eu)	Spain	400	(Eu)	Belgium	13.2%
(Eu)	Spain	177,644	(Eu)	Switzerland	3043	(Eu)	Italy	21,645	(Eu)	Belgium	383	(Eu)	Italy	13.1%
(Eu)	Italy	165,155	(Eu)	Belgium	2897	(Eu)	Spain	18,708	(Eu)	Italy	358	(Eu)	UK	13.1%
(Eu)	France	147,863	(Eu)	Italy	2732	(Eu)	France	17,167	(Eu)	France	263	(Eu)	France	11.6%
(Eu)	Germany	133,456	(Eu)	Ireland	2,541	(Eu)	UK	12,868	(Eu)	UK	190	(Eu)	Netherlands	11.1%
(Eu)	UK	98,476	(Eu)	France	2265	(As)	Iran	4777	(Eu)	Netherlands	183	(Eu)	Spain	10.5%
(As)	** China	82,295	(NA)	USA	1938	(Eu)	Belgium	4440	(Eu)	Switzerland	143	(Eu)	Sweden	10.1%
(As)	Iran	76,389	(Eu)	Portugal	1774	(Eu)	Germany	3592	(Eu)	Sweden	119	(Eu)	Hungary	8.6%
(As)	Turkey	69,392	(Eu)	Netherlands	1643	(As)	** China	3342	(Eu)	Ireland	90	(As)	Iran	6.3%
(Eu)	Belgium	33,573	(Eu)	Germany	1593	(Eu)	Netherlands	3134	(NA)	USA	86	(SA)	Brazil	6.0%
(SA)	Brazil	28,320	(Eu)	Austria	1592	(SA)	Brazil	1736	(Eu)	Portugal	59	(Eu)	Albania	5.2%
(NA)	Canada	28,205	(Eu)	UK	1451	(As)	Turkey	1518	(As)	Iran	57	(NA)	Dominican R.	5.1%
(Eu)	Netherlands	28,153	(As)	Israel	1444	(Eu)	Switzerland	1239	(Eu)	Denmark	53	(Eu)	Romania	5.1%
(Eu)	Switzerland	26,336	(As)	Qatar	1288	(Eu)	Sweden	1203	(Eu)	Austria	44	(SA)	Ecuador	4.9%
(Eu)	Russia	24,490	(Eu)	Norway	1243	(NA)	Canada	1006	(Eu)	Germany	43	(Eu)	Slovenia	4.8%
(Eu)	Portugal	18091	(Eu)	Sweden	1181	(Eu)	Portugal	599	(Eu)	Slovenia	29	(Eu)	Greece	4.8%
(Eu)	Austria	14,336	(Eu)	Denmark	1153	(As)	Indonesia	469	(Eu)	Norway	28	(Eu)	N. Macedonia	4.7%
(Eu)	Ireland	12,547	(Eu)	Estonia	1055	(Eu)	Ireland	444	(NA)	Canada	27	(Eu)	Switzerland	4.7%
(As)	Israel	12,501	(As)	Bahrain	982	(NA)	Mexico	406	(Eu)	Estonia	26	(Eu)	Bulgaria	4.6%
(As)	India	12322	(As)	Iran	909	(As)	India	405	(NA)	Panama	22	(Eu)	Denmark	4.6%
(Eu)	Sweden	11,927	(NA)	Panama	828	(Eu)	Austria	393	(Eu)	N. Macedonia	22	(NA)	USA	4.4%
(SA)	Peru	11,475	(As)	Turkey	823	(SA)	Ecuador	388	(SA)	Ecuador	22	(Eu)	Poland	4.0%
(As)	S. Korea	10,591	(NA)	Canada	747	(Eu)	Romania	372	(Eu)	Romania	19	(NA)	Canada	3.6%
(SA)	Chile	8273	(As)	Singapore	632	(As)	Philippines	349	(As)	Turkey	18	(Eu)	Bosnia-Herzeg.	3.6%
(As)	Japan	8100	(Eu)	Slovenia	600	(Af)	Algeria	336	(NA)	Dominican Rep.	17	(Eu)	Ireland	3.5%
(SA)	Ecuador	7858	(As)	Cyprus	592	(Eu)	Denmark	309	(Eu)	Czechia	16	(Eu)	Portugal	3.3%
(Eu)	Poland	7582	(Eu)	Finland	584	(Eu)	Poland	286	(As)	Israel	15	(Eu)	Austria	2.8%
(Eu)	Romania	7216	(Eu)	Czechia	580	(SA)	Peru	254	(Eu)	Hungary	14	(Eu)	Czechia	2.8%
(Eu)	Norway	6740	(Eu)	Serbia	558	(As)	S. Korea	225	(Eu)	Finland	13	(Af)	Mauritius	2.7%
(Eu)	Denmark	6681	(As)	UAE	542	(Eu)	Russia	198	(Eu)	Bosnia-Herzeg.	12	(Eu)	Lithuania	2.7%
(AO)	Australia	6447	(Eu)	Moldova	508	(NA)	Dominican R.	189	(Eu)	Lithuania	11	(Eu)	Germany	2.7%
(As)	Pakistan	6,383	(Eu)	N. Macedonia	468	(Af)	Egypt	183	(Eu)	Moldova	11	(NA)	Panama	2.7%
(Eu)	Czechia	6216	(SA)	Ecuador	445	(Eu)	Czechia	166	(Eu)	Serbia	11	(Eu)	Estonia	2.5%
(As)	Saudi Arabia	5862	(SA)	Chile	433	(Eu)	Norway	150	(As)	Cyprus	10	(SA)	Peru	2.3%
(As)	Philippines	5453	(Eu)	Croatia	424	(As)	Japan	146	(Eu)	Greece	10	(Eu)	Norway	2.3%
(NA)	Mexico	5399	(Eu)	Lithuania	401	(Eu)	Hungary	134	(Eu)	Albania	9	(Eu)	Finland	2.2%
(As)	UAE	5365	(Eu)	Belarus	395	(As)	Israel	130	(Eu)	Croatia	8	(As)	Turkey	2.2%
(As)	Indonesia	5136	(As)	Armenia	375	(Af)	Morocco	127	(Af)	Algeria	8	(Eu)	Moldova	2.2%
(As)	Malaysia	5072	(Eu)	Romania	375	(SA)	Colombia	127	(Eu)	Poland	8	(Eu)	Serbia	2.0%
(Eu)	Serbia	4873	(Eu)	Latvia	353	(SA)	Argentina	111	(SA)	Peru	8	(As)	S. Korea	1.9%
(Eu)	Ukraine	3764	(SA)	Peru	348	(As)	Pakistan	111	(SA)	Brazil	8	(As)	Malaysia	1.9%
(Eu)	Belarus	3728	(Eu)	Bosnia-Herzeg.	338	(Eu)	Ukraine	108	(Af)	Mauritius	7	(Eu)	Croatia	1.9%
(As)	Qatar	3711	(NA)	Dominican R.	333	(Eu)	Greece	102	(NA)	Trinidad-Tobago	6	(As)	Cyprus	1.7%
(As)	Singapore	3699	(As)	Kuwait	329	(Eu)	Serbia	99	(As)	Armenia	6	(As)	Armenia	1.6%
(NA)	Dominican R.	3614	(AO)	New Zealand	287	(NA)	Panama	95	(Eu)	Bulgaria	5	(SA)	Uruguay	1.4%
(NA)	Panama	3574	(Af)	Mauritius	255	(SA)	Chile	94	(SA)	Chile	5	(As)	Saudi Arabia	1.2%
(Eu)	Finland	3237	(AO)	Australia	253	(As)	Malaysia	83	(As)	Bahrain	4	(SA)	Chile	1.2%
(SA)	Colombia	2979	(Eu)	Greece	210	(As)	Iraq	79	(Eu)	Belarus	4	(As)	Israel	1.0%
(As)	Thailand	2643	(As)	S. Korea	207	(As)	Saudi Arabia	79	(As)	S. Korea	4	(Eu)	Belarus	1.0%
(Af)	S. Africa	2506	(Eu)	Poland	200	(Eu)	Finland	72	(Eu)	Latvia	3	(Eu)	Latvia	0.8%
(Af)	Egypt	2505	(As)	Oman	178	(AO)	Australia	63	(NA)	Honduras	3	(As)	Azerbaijan	0.8%
(SA)	Argentina	2443	(Eu)	Albania	172	(Eu)	Slovenia	61	(As)	Lebanon	3	(AO)	Australia	0.8%
(Eu)	Greece	2192	(As)	Saudi Arabia	168	(As)	Bangladesh	50	(Af)	Tunisia	3	(AO)	New Zealand	0.7%
(Af)	Algeria	2160	(Eu)	Russia	168	(Eu)	Moldova	46	(Af)	Morocco	3	(NA)	Costa Rica	0.7%
(Eu)	Moldova	2049	(Eu)	Hungary	163	(Eu)	N. Macedonia	45	(As)	UAE	3	(Eu)	Slovakia	0.6%
(Af)	Morocco	2024	(Eu)	Slovakia	158	(As)	Thailand	43	(As)	Malaysia	3	(Eu)	Russia	0.6%

**Table 2:** Monthly mean weather indexes for Wuhan, Milan, Bergamo and Brescia in 2020.

	**Mean Monthly Temp. (°C)**			**Min Daily Temp. (°C)**			**Max Daily Temp. (°C)**		
	**January**	**February**	**March**	**January**	**February**	**March**	**January**	**February**	**March**
Wuhan	4.1±3	8.4±3.6	12.9±4.1	1	3	6	8	18	19
Milan	3.8±2.0	8.1±2.1	9.5±2.8	0	4	5	7	13	15
Bergamo	4.3±1.6	7.7±1.7	8.8±2.9	1	5	4	7	11	14
Brescia	3.2±2.0	7.2±1.6	8.7±2.4	−1	4	5	7	10	14
	**Relative Humidity (%)**			**Wind Speed (km/h)**			**Pressure (mbar)**		
	**January**	**February**	**March**	**January**	**February**	**March**	**January**	**February**	**March**
Wuhan	74	66	66	10	10	11	1025	1024	1018
Milan	85±8	67±19	68±11	5±1	8±4	8±3	1026±7	1020±8	1017±8
Bergamo	76±10	61±20	68±11	6±1	9±3	8±2	1026±7	1019±8	1016±8
Brescia	85±7	72±16	77±9	6±2	8±4	10±5	1025±7	1018±8	1016±8

**Table 3:** Total cases and deaths, deaths per million people, deaths per cases and weather means for March 2020 for each Italian region. Regional population density and mean age are added for comparison. The regions are ranked by the number of deaths per million people. (N) Northern Italy; (C) Central Italy; (S) Southern Italy. The Pearson correlation coefficients are relative to the logarithm of Deaths/1Mil.

Region	Total	Total	Deaths	Deaths	Temp.	RH	Wind	Density	Mean
	Cases	Deaths	/1Mil.	/Cases	(°C)	(%)	km/h	pop/km^2^	Age (y)
(N) Lombardia	62,153	11,377	1131	18.3%	9.2	69	9	422	44.3
(N) Valle d’Aosta	958	121	963	13%	8.4	70	9	39	45.1
(N) Emilia-Romagna	21,029	2788	625	13.3%	9.7	75	10	199	45.4
(N) Liguria	5936	807	520	13.6%	11.9	62	15	286	48.2
(N) Trentino-A. A.	5444	541	505	9.9%	9.8	56	6	79	42.8
(C) Marche	5503	746	489	13.6%	9.5	72	12	162	45.6
(N) Piemonte	18,229	2015	463	11.1%	9.3	66	8	172	46.1
(N) Veneto	14,624	940	192	6.4%	10.0	67	8	267	44.6
(C) Abruzzo	2274	240	183	10.6%	10.0	72	12	121	45.2
(N) Friuli Venezia G.	2544	212	174	8.3%	10.1	62	11	153	46.6
(C) Toscana	7666	556	149	7.3%	10.7	71	11	162	46.2
(S) Puglia	3184	288	71	9.0%	11.6	76	14	206	43.6
(C) Umbria	1322	54	61	4.1%	9.0	72	13	104	46.0
(S) Molise	263	15	49	5.7%	7.7	74	13	69	45.8
(C) Lazio	5232	311	53	5.9%	12.4	64	12	341	44.0
(S) Sardegna	1161	83	51	7.1%	12.4	76	13	68	45.6
(S) Campania	3807	278	48	7.3%	13.1	63	12	424	41.6
(S) Calabria	971	71	36	7.3%	12.5	75	15	128	43.4
(S) Sicilia	2535	181	36	7.1%	13.2	73	15	194	43.0
(S) Basilicata	320	21	37	6.6%	9.4	76	14	56	44.7
Pearson Corr. (r)			log( x)		−0.49	−0.37	−0.67	0.12	0.34

**Table 4:** Number of deaths per million people, and weather temperature and relative humidity means for March 2020 for each state of the USA. Median population age is added for comparison. The Pearson correlation coefficients in the parentheses exclude the State of Louisiana. See text for details.

State	Largest City	Cases/1Mil	Deaths/1Mil	Deaths/Cases	Mean Tem. (°C)	RH (%)	Median Age (y)
Alabama	Birmingham	853	25	2.9%	17	73	39.2
Alaska	Anchorage	397	12	3.0%	−8	88	34.6
Arizona	Phoenix	570	20	3.5%	19	38	37.9
Arkansas	Little Rock	525	11	2.1%	15	77	38.3
California	Los Angeles	682	22	3.2%	17	57	36.8
Colorado	Denver	1436	59	4.1%	9	50	36.9
Connecticut	Bridgeport	4120	242	5.9%	7	67	41.0
Delaware	Wilmington	2028	45	2.2%	10	66	40.7
Dis. of Columbia	Washington	3210	105	3.3%	12	65	34.0
Florida	Jacksonville	1093	29	2.7%	24	64	42.2
Georgia	Atlanta	1455	54	3.7%	17	70	36.9
Hawaii	Honolulu	364	6	1.6%	23	77	39.2
Idaho	Boise	867	23	2.7%	6	60	36.6
Illinois	Chicago	1918	74	3.9%	5	72	38.3
Indiana	Indianapolis	1349	66	4.9%	9	71	37.9
Iowa	Des Moines	637	17	2.7%	7	74	38.2
Kansas	Wichita	514	26	5.1%	12	64	36.9
Kentucky	Louisville	498	26	5.2%	11	71	38.9
Louisiana	New Orleans	4707	237	5.0%	23	73	37.2
Maine	Portland	578	18	3.1%	4	69	44.9
Maryland	Baltimore	1671	58	3.5%	11	65	38.8
Massachusetts	Boston	4380	162	3.7%	7	60	39.4
Michigan	Detroit	2818	193	6.8%	6	69	39.8
Minnesota	Minneapolis	327	16	4.9%	3	73	38.1
Mississippi	Jackson	1124	41	3.6%	19	80	37.7
Missouri	Kansas City	804	24	3.0%	11	71	38.7
Montana	Billings	388	7	1.8%	3	62	39.9
Nebraska	Omaha	473	11	2.3%	8	70	36.6
Nevada	Las Vegas	1099	44	4.0%	14	44	38.1
New Hampshire	Manchester	812	20	2.5%	6	69	43.0
New Jersey	Newark	7997	355	4.4%	9	61	40.0
New Mexico	Albuquerque	709	17	2.4%	11	39	38.1
New York	New York City	10,897	591	5.4%	8	69	39.0
North Carolina	Charlotte	519	13	2.5%	15	73	38.9
North Dakota	Fargo	485	12	2.5%	−3	90	35.2
Ohio	Columbus	669	31	4.6%	9	69	39.4
Oklahoma	Oklahoma City	578	31	5.4%	15	65	36.7
Oregon	Portland	407	14	3.4%	9	67	39.4
Pennsylvania	Philadelphia	2092	61	2.9%	11	61	40.8
Rhode Island	Providence	3340	82	2.5%	7	67	40.1
South Carolina	Charleston	738	22	3.0%	19	76	39.6
South Dakota	Sioux Falls	1351	7	0.5%	4	76	37.1
Tennessee	Nashville	914	20	2.2%	14	71	38.8
Texas	Houston	556	13	2.3%	23	71	34.8
Utah	Salt Lake City	835	7	0.8%	7	59	31.0
Vermont	Burlington	1214	48	4.0%	2	78	42.8
Virginia	Virginia Beach	773	23	3.0%	13	73	38.4
Washington	Seattle	1480	75	5.1%	8	73	37.7
West Virginia	Charleston	384	5	1.3%	11	78	42.7
Wisconsin	Milwaukee	644	31	4.8%	4	75	39.6
Wyoming	Cheyenne	493	2	0.4%	5	59	38.0
USA	mean	1545	62	3.3%	10.3	68	38.2
Pearson Corr. (r)		log(x)			−0.29 (−0.40)	−0.29 (−0.30)	0.10 (0.12)
Pearson Corr. (r)			log(x)		−0.23 (−0.34)	−0.27 (−0.28)	0.14 (0.17)

**Table 5:** World distribution of COVID-19 cases and deaths with relative statistics per million people for 15 April 2020.

	Total	Total	Total	Active	Cases	Deaths	Deaths	Population
	Cases	Deaths	Recovered	Cases	/1Mil	/1Mil	/Cases	
Europe	966,268	88,003	257,966	620,299	1292	118	9.1%	747,636,026
North America	685,604	30,230	60,102	595,272	1158	51	4.4%	592,072,212
South America	62,750	2770	22,268	37,712	146	6.4	4.4%	430,759,766
Asia	332,556	12,019	159,944	160,593	72	2.6	3.6	4,641,054,775
Oceania	7924	72	4415	3437	186	1.7	0.9%	42,677,813
Africa	17,745	912	3733	13,100	13	0.7	5%	1,340,598,147
World	2,073,568	134,020	509,067	1,430,481	266	17.2	6.5%	7,794,798,739

**Table 6:** Boxplot data: see Figure 8.

	**Cases/1Mil**		
	***T* < 2 °C**	**2 °C < *T* < 14 °C**	***T* > 14 °C**
maximum	1243	3043	142
Q3	584	1592	60
median	375	468	23
Q1	77	158	4
minimum	9	0.5	0
	**Deaths/1Mil**		
	***T* < 2 °C**	**2 °C < *T* < 14 °C**	***T* > 14 °C**
maximum	27	119	4
Q3	13	59	2
median	4	14	0.4
Q1	0.9	4	0.1
minimum	0	0	0
	**Deaths/Cases**		
	***T* < 2 °C**	**2 °C < *T* < 14 °C**	***T* > °C**
maximum	3.6	11.6	5.1
Q3	2.5	6.3	2.3
median	2.2	4.6	1.0
Q1	1.0	2.7	0.4
minimum	0.6	0.6	0.2

**Table 7:** Sample of the COVID-19 data ranked by the country mean population age. The complete table is found in the Supplementary Materials.

		Median	Cases	Deaths	Deaths	March	March			Median	Cases	Deaths	Deaths	March	March
#	Country	Age (y)	/1Mil	/1Mil	/Cases	T (°C)	RH (%)	#	Country	Age (y)	/1Mil	/1Mil	/Cases	T (°C)	RH (%)
1	Japan	48.6	64	1	1.6 %	4.7	73	31	Belgium	41.6	2897	383	13.2%	6.6	79
2	Germany	47.8	1593	43	2.7%	4.9	77	32	Ukraine	41.2	86	2	2.3 %	2.8	76
3	Italy	46.5	2732	358	13.1%	9.5	73	33	Sweden	41.1	1181	119	10.1%	−3.3	88
4	Hong Kong	45.6	136	0.5	0.4%	19.4	78	34	Belarus	40.9	395	4	1.0%	0.8	77
5	Greece	45.3	210	10	4.8 %	10.9	68	35	United Kingdom	40.6	1451	190	13.1%	5.7	84
6	Slovenia	44.9	600	29	4.8 %	5.7	69	36	Russia	40.3	168	1	0.6%	−13.2	96
7	Portugal	44.6	1774	59	3.3 %	12.4	70	37	Norway	39.5	1243	28	2.3 %	−3.5	80
8	Austria	44.5	1592	44	2.8 %	2.8	73	38	Macedonia	39	468	22	4.7 %	5.9	61
9	Lithuania	44.5	401	11	2.7 %	0.9	78	39	Thailand	39	38	0.6	1.6 %	27.8	67
10	Latvia	44.4	353	3	0.8 %	0.0	80	40	Georgia	38.6	77	0.8	1.0 %	0.4	63
11	Croatia	43.9	424	8	1.9 %	7.4	68	41	USA	38.5	1938	86	4.4%	3.2	74
12	Spain	43.9	3799	400	10.5%	9.9	65	42	China	38.4	57	2	3.5 %	2.6	61
13	Bulgaria	43.7	108	5	4.6 %	6.2	68	43	U. Arab Emirates	38.4	542	3	0.6 %	23.6	59
14	Estonia	43.7	1055	26	2.5 %	−0.9	83	44	Cyprus	37.9	592	10	1.7 %	14.1	68
15	Hungary	43.6	163	14	8.6 %	6.2	71	45	Ireland	37.8	2541	90	3.5%	6.7	83
16	Serbia	43.4	558	11	2.0 %	6.2	68	46	Trinidad and Tob.	37.8	81	6	7.4 %	25.7	78
17	Bosnia-Her.	43.3	338	12	3.6%	6.1	67	47	Moldova	37.7	508	11	2.2%	4.2	72
18	Czech Republic	43.3	580	16	2.8%	3.7	74	48	Australia	37.5	253	2	0.8 %	25.7	51
19	South Korea	43.2	207	4	1.9 %	5.8	62	49	New Zealand	37.2	287	2	0.7 %	14.1	80
20	Finland	42.8	584	13	2.2%	-4.7	84	50	Armenia	36.6	375	6	1.6 %	1.9	52
21	Netherlands	42.8	1643	183	11.1 %	6.2	81	51	Mauritius	36.3	255	7	2.7 %	25.6	83
22	Switzerland	42.7	3043	143	4.7%	2.2	73	52	Singapore	35.6	632	2	0.3 %	27.6	85
23	Romania	42.5	375	19	5.1%	4.6	73	53	Chile	35.5	433	5	1.2	11.4	67
24	Taiwan	42.3	17	0.3	1.8 %	21	79	54	Uruguay	35.5	142	2	1.4 %	21.6	73
25	Cuba	42.1	72	2	2.8 %	24.5	75	55	Albania	34.3	172	9	5.2 %	7.5	64
26	Denmark	42	1153	53	4.6 %	2.9	85	56	Lebanon	33.7	96	3	3.1%	12.5	69
27	Poland	41.9	200	8	4.0%	3.6	75	57	Qatar	33.7	1288	2	0.2%	23.3	56
28	Canada	41.8	747	27	3.6 %	−15.5	100	58	Sri Lanka	33.7	11	0.3	2.7%	28.0	75
29	Slovakia	41.8	158	1	0.6 %	2.8	73	59	Brazil	33.2	133	8	6.0%	26.1	83
30	France	41.7	2265	263	11.6%	7.8	74	60	Bahrain	32.9	982	4	0.4 %	23.2	52

**Table 8:** Air pollution world country index arranged by average PM2.5 concentration (μg/m^3^), weighted by population. Source: IQAir, 2019 World Air Quality Report (https://www.iqair.com/, accessed on 15 April 2020).

Rank	Country	AQI	Rank	County	AQI	Rank	Country	AQI
1	Bangladesh	83.3	34	Laos	23.1	67	Lithuania	14.5
2	Pakistan	65.8	35	Chile	22.6	68	Czech Republic	14.5
3	Mongolia	62	36	Greece	22.5	69	Latvia	13.3
4	Afghanistan	58.8	37	Saudi Arabia	22.1	70	Belgium	12.5
5	India	58.1	38	South Africa	21.6	71	France	12.3
6	Indonesia	51.7	39	Nigeria	21.4	72	Austria	12.2
7	Bahrain	46.8	40	Algeria	21.2	73	Japan	11.4
8	Nepal	44.5	41	Cambodia	21.1	74	Germany	11
9	Uzbekistan	41.2	42	Israel	20.8	75	Netherlands	10.9
10	Iraq	39.6	43	Turkey	20.6	76	Switzerland	10.9
11	China Mainland	39.1	44	Hong Kong	20.3	77	Ireland	10.6
12	United Arab Emirates	38.9	45	Guatemala	20.2	78	United Kingdom	10.5
13	Kuwait	38.3	46	Ethiopia	20.1	79	Costa Rica	10.4
14	Bosnia & Herzegovina	34.6	47	Georgia	20.1	80	Puerto Rico	10.2
15	Vietnam	34.1	48	Mexico	20	81	Russia	9.9
16	Kyrgyzstan	33.2	49	Cyprus	19.7	82	Spain	9.7
17	North Macedonia	32.4	50	Malaysia	19.4	83	Luxembourg	9.6
18	Syria	32.2	51	Croatia	19.1	84	Denmark	9.6
19	DR Congo	32.1	52	Singapore	19	85	Malta	9.4
20	Myanmar	31	53	Poland	18.7	86	Portugal	9.3
21	Ghana	30.3	54	Romania	18.3	87	USA	9
22	Uganda	29.1	55	Jordan	18.3	88	Ecuador	8.6
23	Armenia	25.5	56	Egypt	18	89	Australia	8
24	Bulgaria	25.5	57	Philippines	17.6	90	Canada	7.7
25	Sri Lanka	25.2	58	Taiwan	17.2	91	New Zealand	7.5
26	South Korea	24.8	59	Italy	17.1	92	Norway	6.9
27	Iran	24.3	60	Ukraine	16.6	93	Sweden	6.6
28	Thailand	24.3	61	Slovakia	16.1	94	Estonia	6.2
29	Kazakhstan	23.6	62	Angola	15.9	95	Finland	5.6
30	Kosovo	23.5	63	Brazil	15.8	96	Iceland	5.6
31	Macao SAR	23.5	64	Colombia	14.6	97	U.S. Virgin Islands	3.5
32	Serbia	23.3	65	Argentina	14.6	98	Bahamas	3.3
33	Peru	23.3	66	Hungary	14.6			

**Figure A1:**
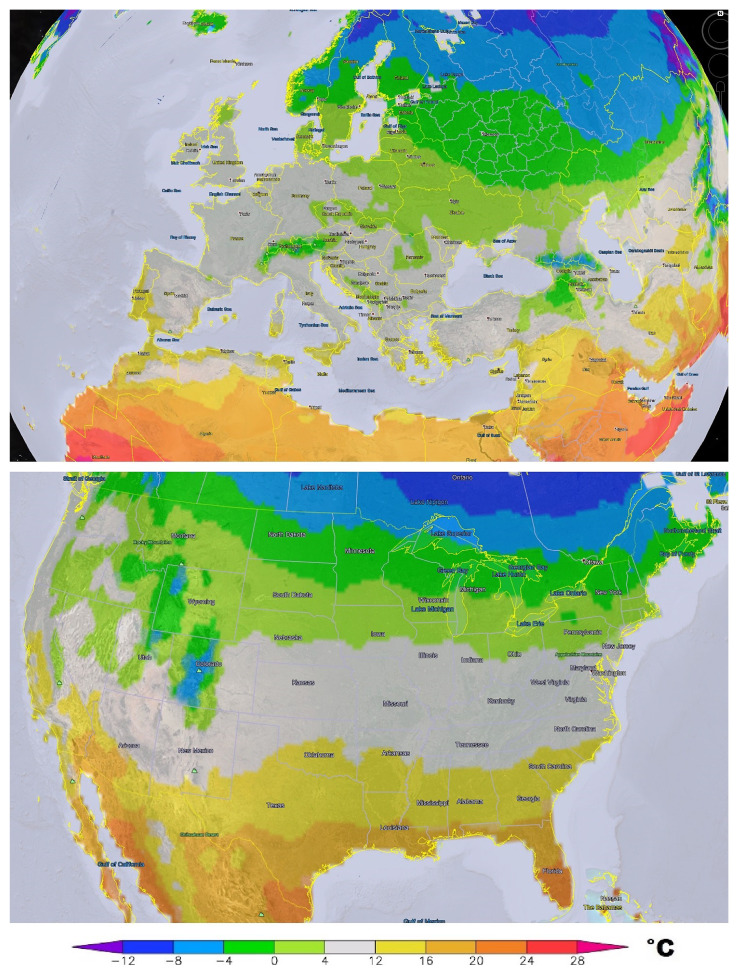
Examples of isotherm Google-Earth-Pro interactive-maps provided as online supplementary files. The light-gray zone represents the modeled most exposed regions to the COVID-19 pandemic. The figure shows the maps of Europe and in the Unites States of America for March.

## Supplementary Materials

The following are available online at https://www.mdpi.com/1660-4601/17/10/3493/s1.

## Appendix A

The online Supplementary files provide MS Excel Tables with all analyzed data and the same twelve isotherm maps shown in Figure 11, Figure 12, Figure 13 and Figure 14 for each month of the year from January to December as Keyhole Markup language Zipped (kmz) files, that is, as Climate Explorer Google-Earth-Pro interactive and zoomable maps. Figure A1 shows an example of the produced maps. Google Earth Pro (used version: 7.3) or equivalent Earth Viewer software is required to visualize the files. (Web site: https://www.google.com/earth/, accessed on 15 April 2020.)
