# Supplementary material for: Distribution of the SARS-CoV-2 Pandemic and Its Monthly Forecast Based on Seasonal Climate Patterns
Source: Int J Environ Res Public Health. 2020 May 17;17(10):3493. doi: 10.3390/ijerph17103493 (PMC7277369; doi:10.3390/ijerph17103493)
Supplement: Supplementary file 1 [file ijerph-17-03493-s001.zip › Supplementary_Files/ReadMeFirst_Supplementary_Files.pdf]

Supplementary Files to "A proposal for isotherm world maps to forecast the seasonal evolution of the SARS-CoV-2 pandemic" Int. J. Environ. Res. Public Health, 2020.

Nicola Scafetta

May 16, 2020

Department of Earth Sciences, Environment and Georesources, University of Naples Federico II, Via Cinthia 21, 80126, Naples, Italy
Tel.: +39 081-2538348
Email: nicola.scafetta@unina.it
ORCID ID: 0000-0003-0967-1911

The online Supplementary Files provides the full date set analyzed in the paper and the same twelve isotherm maps shown in Figures 8, 9, 10 e 11 shown in the main paper for each month of the year from January to December as Keyhole Markup language Zipped (kmz) files, that is, as Climate Explorer Google-Earth-Pro interactive and zoomable maps.

List of provided files:

- 1_January Climate Explorer map.kmz
- 2_February Climate Explorer map.kmz
- 3_March Climate Explorer map.kmz
- 4_April Climate Explorer map.kmz
- 5_May Climate Explorer map.kmz
- 6_June Climate Explorer map.kmz
- 7_July Climate Explorer map.kmz
- 8_August Climate Explorer map.kmz
- 9_September Climate Explorer map.kmz
- 10_October Climate Explorer map.kmz
- 11_November Climate Explorer map.kmz
- 12_December Climate Explorer map.kmz

Google Earth Pro (used version: 7.3: Web site: https://www.google.com/earth/, accessed on 04/01/2020) or equivalent Earth Viewer software is required to visualize the files.

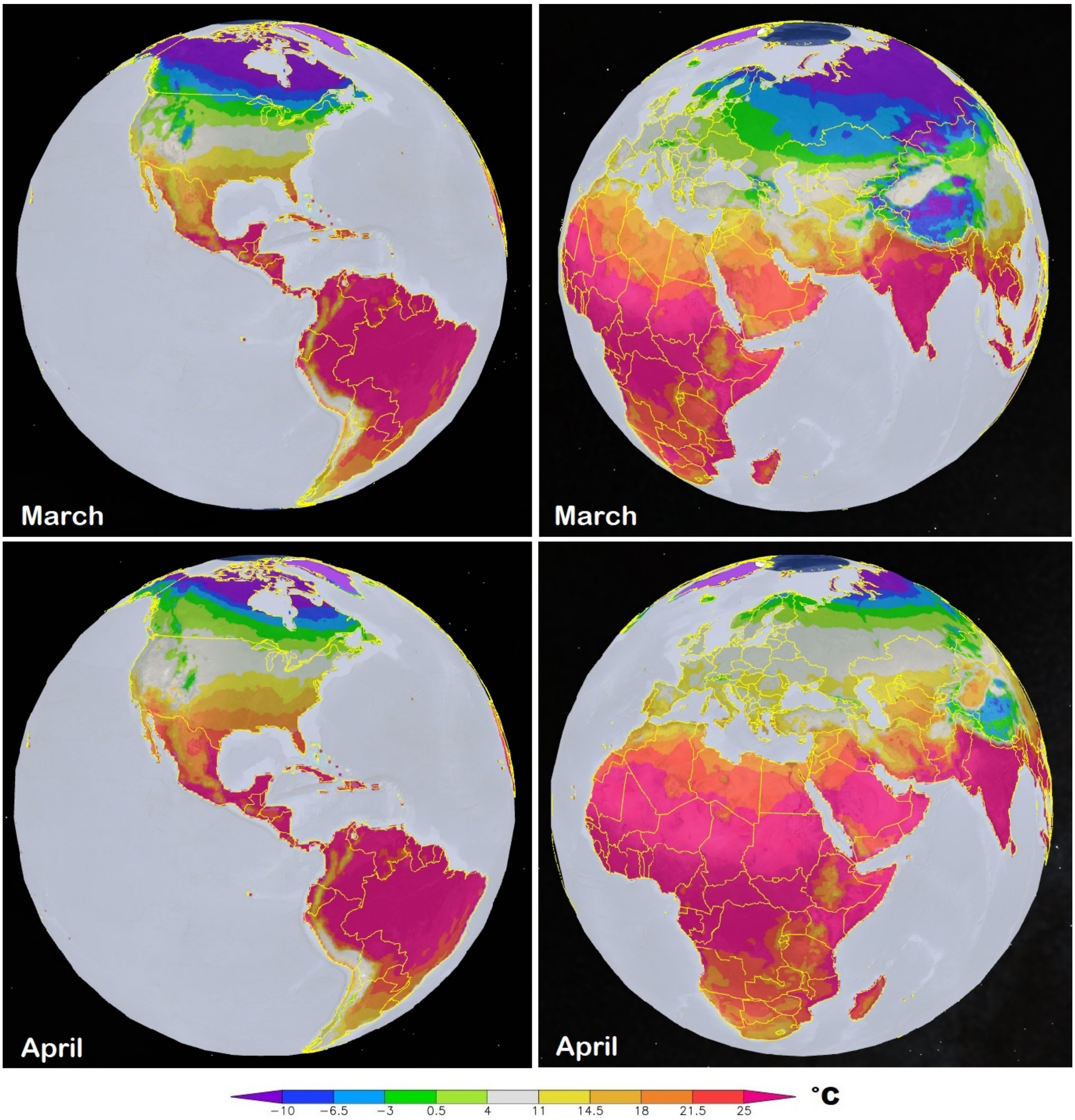

Figure 1: Examples of isotherm Google-Earth-Pro interactive-maps provided as online Supplement Files.
